# Mechanochemical synthesis of poly(trimethylene carbonate)s: an example of rate acceleration

**DOI:** 10.3762/bjoc.15.93

**Published:** 2019-04-23

**Authors:** Sora Park, Jeung Gon Kim

**Affiliations:** 1 Department of Chemistry and Research Institute of Physics and Chemistry, Chonbuk National University, Jeon-Ju, Jeollabuk-do, 54896, Republic of Korea

**Keywords:** aliphatic polycarbonate, green polymerization, mechanochemistry, organocatalyst, poly(trimethylene carbonate)

## Abstract

Mechanochemical polymerization is a rapidly growing area and a number of polymeric materials can now be obtained through green mechanochemical synthesis. In addition to the general merits of mechanochemistry, such as being solvent-free and resulting in high conversions, we herein explore rate acceleration under ball-milling conditions while the conventional solution-state synthesis suffer from low reactivity. The solvent-free mechanochemical polymerization of trimethylene carbonate using the organocatalysts 1,8-diazabicyclo[5.4.0]undec-7-ene (DBU) and 1,5,7-triazabicyclo[4.4.0]dec-5-ene (TBD) are examined herein. The polymerizations under ball-milling conditions exhibited significant rate enhancements compared to polymerizations in solution. A number of milling parameters were evaluated for the ball-milling polymerization. Temperature increases due to ball collisions and exothermic energy output did not affect the polymerization rate significantly and the initial mixing speed was important for chain-length control. Liquid-assisted grinding was applied for the synthesis of high molecular weight polymers, but it failed to protect the polymer chain from mechanical degradation.

## Introduction

Nowadays mechanochemical syntheses are widespread in many areas of chemistry [1–4]. The efficient mixing and energy input induced by mechanical motions have promoted many chemical reactions with superior efficiencies [5]. Sometimes, unexpected outcomes that cannot be achieved by solution synthesis occur, which makes mechanochemistry a topic of rigorous research [6].

In the area of polymer chemistry, the use of mechanical forces has a long history. Strong mechanical forces can break covalent bonds, including strong C–C bonds, thus their utilization has generally focused on destructive approaches [7–9]. Recently, along with rapid progress in mechanochemical small molecule syntheses, the constructive polymeric material synthesis also succeeded. In 2014, Swager and co-workers demonstrated that poly(phenylene vinylene) could be obtained without any solvent after brief ball milling of monomer and base [10]. The remarkable reactivity exemplified that the general concepts of mechanochemical synthesis are applicable to polymerization reactions. Other examples of polymer syntheses have followed. The Borchardt research team reported the efficient mechanochemical synthesis of poly(azomethine) and poly(phenylene) [11–12]. Our group also contributed to this area by developing a ball-milling promoted high-molecular weight poly(lactic acid) synthesis [13–14] and a solvent-free post-polymerization modification of functional polystyrenes [15]. The Friščić team also showed that poly(ethylene oxide) end group modification is facile under ball-mill conditions [16]. Network polymeric material fabrications were also realized using ball milling [17–19].

**Scheme 1:**
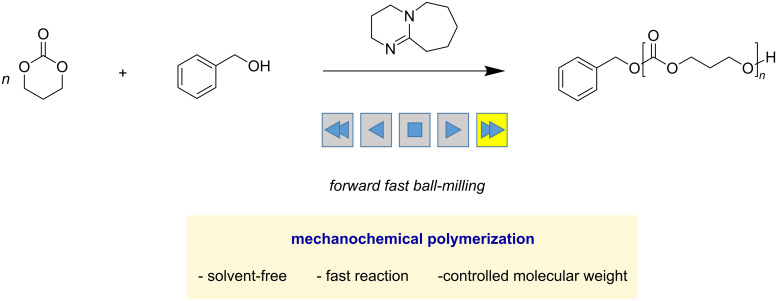
Fast trimethylene carbonate polymerization using a solvent-free ball-milling approach.

As mentioned, many mechanochemical reactions realized exceptional efficiencies that solution synthesis cannot afford [5,20]. Chemical transformations at maximum concentrations benefit from no dilution, which results in fast conversions, as long as efficient mixing is provided. We envisioned that polymerization systems with low propagation efficiencies under solution conditions could be accelerated through mechanochemical ball milling. The organocatalytic polymerization of trimethylene carbonate to form aliphatic polycarbonates was found to be more efficient when using a mechanical ball-milling reaction than a solution polymerization (Scheme 1). The detailed findings are disclosed in this article.

## Results and Discussion

Aliphatic polycarbonates are found in many biomedical applications since they have many desirable properties such as high biocompatibility, easy degradation, good mechanical properties, and low toxicity [21–23]. Many synthetic methods have been developed, and the chain-growth ring-opening polymerization of cyclic carbonates, such as trimethylene carbonate (TMC) and its derivatives have been used for the controlled synthesis of high-molecular weight polymers. Among many catalysts, organocatalysts have attracted considerable attention, since the use of nontoxic catalysts warrants a safe use in biomedical applications [24].

The amidine base 1,8-diazabicyclo[5.4.0]undec-7-ene (DBU) is one of the best studied and most popular organocatalysts for ring-opening polymerizations of cyclic carbonates and lactones [25–27]. In contrast to the high activity of lactone polymerization, cyclic carbonate polymerization usually requires long reaction times to achieve high conversions (Table 1) [27]. The DBU-catalyzed polymerization of trimethylene carbonate in chloroform, tetrahydrofuran, toluene, and methylene chloride converted less than 5% monomer into poly(trimethylene carbonate) (PTMC) within 1 h (Table 1, entries 1–8). It generally took 24 hours to produce PTMCs with over 2,000 g/mol number average molecular weights (*M*_n_). Among the tested solvents, the reaction in CH_2_Cl_2_ was the fastest with 43% conversion after 24 h (Table 1, entry 8).

**Table 1:** DBU-catalyzed polymerization of trimethylene carbonate: solution vs ball milling.^a,b^.

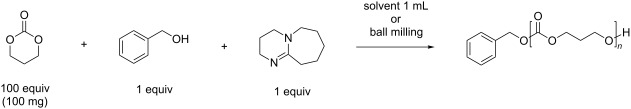						

entry	solvent	time (h)	conv (%)^c^	*M*_n_ (g/mol)^d^	*M*_w_ (g/mol)^d^	*M*_w_/*M*_n_

1	chloroform	1	2	–	–	–
2		24	24	2120	2270	1.07

3	THF	1	<1	–	–	–
4		24	5	–	–	–

5	toluene	1	<1	–	–	–
6		24	23	2660	2950	1.10

7	CH_2_Cl_2_	1	3	–	–	–
8		24	43	3990	4200	1.05

9	ball mill no solvent	0.5	43	3930	4350	1.11
10		1	75	7380	8350	1.14
11		2	93	9230	10600	1.15

^a^Polymerization conditions: TMC (100 mg, 100 equiv), BnOH (1.02 µL, 1 equiv), and DBU (1.46 µL, 1 equiv) in 1 mL of the selected solvent at rt for the solution reactions, or in a 10 mL stainless-steel jar with three 7 mm diameter stainless-steel balls for the ball-milling reactions. ^b^The average of two runs is reported for the ball-milling reactions. ^c^Determined by ^1^H NMR spectroscopy. ^d^Determined by GPC calibrated with polystyrene standards in tetrahydrofuran (THF) at 40 °C.

The same reaction was conducted using ball milling without any solvent added. All reagents were placed in a 10 mL stainless-steel milling jar with three 7 mm diameter stainless-steel balls. The solid-state reaction mixture was placed into a high-speed vibration ball mill. After 30 min of high-speed vibration (30 Hz), 43% conversion was recorded, and PTMC with an *M*_n_ of 3930 g/mol was obtained (Table 1, entry 9), which is comparable to that of PTMC obtained from a 24 h reaction with CH_2_Cl_2_ (Table 1, entry 8). Longer milling times pushed the reaction to higher degrees of polymerization. A one-hour vibration resulted in 75% conversion with an *M*_n_ of 7380 g/mol, and the polymerization reached over 90% conversion after 2 h (Table 1, entries 10 and 11). While the reaction rate was higher than that of the solution reactions, polydispersity under ball-milling conditions remained low (*M*_w_/*M*_n_ = 1.15). To maintain low polydispersity, fast initiation and slow propagation are required [28]. In the case of ball-milling polymerization, the time required for the physical mixing of monomer, catalyst, and initiator would result in a delayed initiation of the polymerization. However, the relatively slow propagation rate of DBU-mediated trimethyl carbonate polymerization allowed for well-controlled chain lengths. The previous examples on mechanochemical poly(lactic acid) synthesis resulted in broader molecular weight distributions due to the fast propagation rate [13–14].

Variations of the ball-milling parameters were then scrutinized (Table 2). Firstly, the vibration frequency was varied from 10 Hz to 30 Hz. Even with the low vibration experiment at 10 Hz for one hour, 60% of trimethylene carbonate were converted into the corresponding polymer (Table 2, entry 1), which is much faster than conversions observed in solution reactions collected in Table 1. An increase in the vibration frequencies exhibited only a marginal effect. At 20 Hz, only 3% increase in conversion was observed (63%, Table 2, entry 2) and at 30 Hz, 75% conversion was recorded (Table 2, entry 3). The effect of vibration frequency was found to be less pronounced as in the case of lactide polymerizations [13]. The changes in ball numbers and size were investigated as well, which will increase overall mass of a vibration system. The use of five balls instead of three improved the conversion to 84% and the vibration with a 12 mm ball gave 88% conversion. The mass increase in the vibration system resulted in the improvement of reaction efficiency.

**Table 2:** Vibration frequency and ball size and number effects.^a,b^.

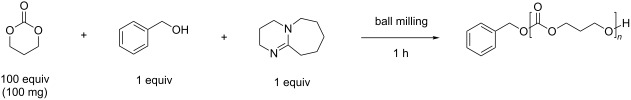					

entry	frequency and ball diameter	conv (%)^c^	*M*_n_ (g/mol)^d^	*M*_w_ (g/mol)^d^	*M*_w_/*M*_n_

1	10 Hz, 7 mm × 3 ea	60	5690	6320	1.11
2	20 Hz, 7 mm × 3 ea	63	6000	6510	1.09
3	30 Hz, 7 mm × 3 ea	75	7380	8350	1.14
4	30 Hz, 7 mm × 5 ea	84	6660	7500	1.13
5	30 Hz, 12 mm × 1 ea	88	6880	7810	1.14

^a^Polymerization conditions: TMC (100 mg, 100 equiv), BnOH (1.02 µL, 1 equiv), and DBU (1.46 µL, 1 equiv) in a 10 mL stainless-steel jar. ^b^The average of the two runs is reported. ^c^Determined by ^1^H NMR spectroscopy. ^d^Determined by GPC calibrated with polystyrene standards in THF at 40 °C.

**Figure 1:**
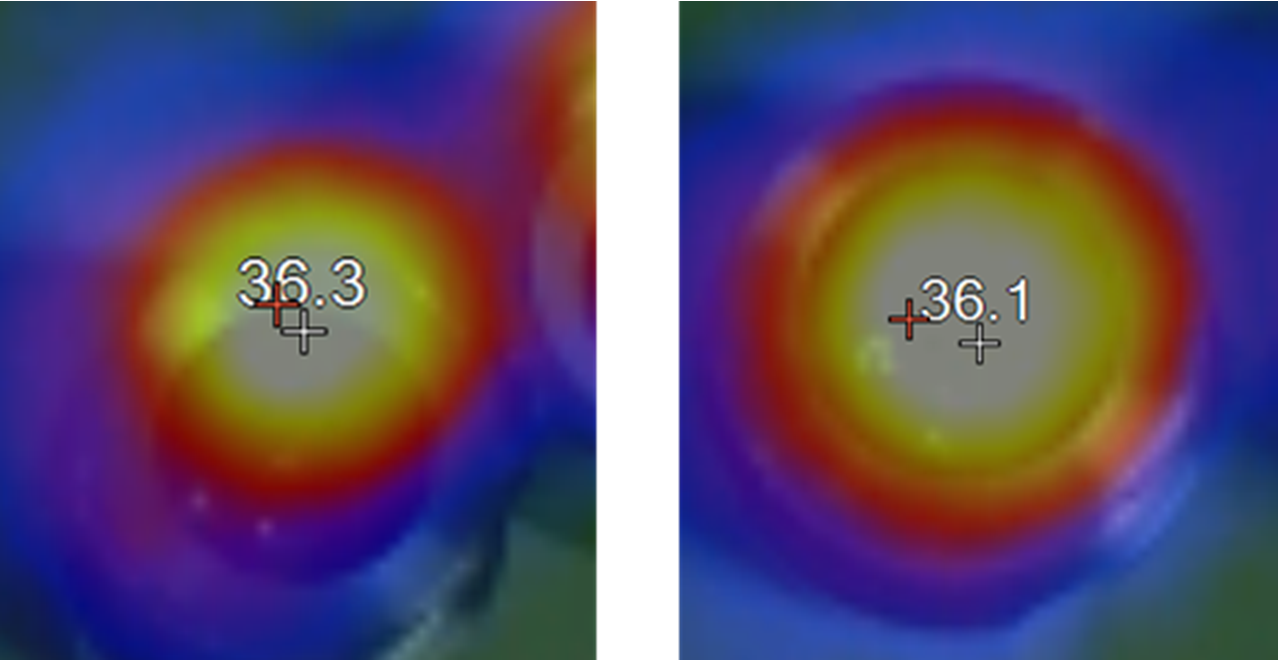
IR thermometer images showing reactor temperatures at the end of the two individual ball-milling reactions (averaged results collected in Table 1, entry 11; individual results, see Supporting Information File 1, left: Table S1, entry 11-1, right: Table S1, entry 11-2).

The high impact collision energy [28–29] and exothermic nature of the given ring-opening polymerization [30] could increase the temperature of a ball-mill system, which would speed up the polymerization rate [31–32]. To gain insight into thermal effects, we monitored the temperature of the reactor and the reaction mixture (Figure 1). After two hours of high-speed ball milling, the temperature of the reactor and mixture increased to 36 °C. To allow a direct comparison, the solution reactions were also conducted at 40 °C. However, their efficiencies remained far behind those of the ball-milling polymerizations (Table 3). In chloroform (Table 3, entries 1 and 2) and toluene (Table 3, entries 5 and 6) rate enhancements by thermal energy were observed, however, their efficiencies remained much lower than that of the ball-milling PTMC synthesis (Table 3, entries 9 and 10). The observed high efficiency of the mechanochemical transformation could originate from a large increase in concentration as well as a temperature difference [5]. In the synthesis of poly(trimethylene carbonate), the observations imply that concentration is a more influential factor than temperature increase for the rate enhancement under ball-milling conditions.

**Table 3:** DBU-catalyzed polymerization of trimethylene carbonate at 40 °C.^a^

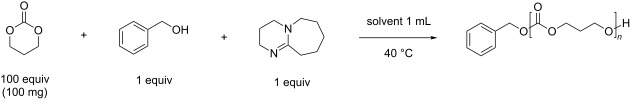						

entry	solvent	time (h)	conv (%)^b^	*M*_n_ (g/mol)^c^	*M*_w_ (g/mol)^c^	*M*_w_/*M*_n_

1	chloroform	1	3	–	–	–
2		24	41	3500	3780	1.07

3	THF	1	<1	–	–	–
4		24	6	–	–	–

5	toluene	1	<1	–	–	–
6		24	70	7130	8380	1.13

7	CH_2_Cl_2_	1	4	–	–	–
8		41	41	3000	3210	1.07

9	ball milling	1	75	7380	8350	1.14

10	ball milling (36 °C)	2	93	9230	10600	1.15

^a^Polymerization conditions: TMC (100 mg, 100 equiv), BnOH (1.02 µL, 1 equiv), and DBU (1.46 µL, 1 equiv) in 1 mL of the selected solvent at 40 °C. ^b^Determined by ^1^H NMR spectroscopy. ^c^Determined by GPC calibrated with polystyrene standards in tetrahydrofuran (THF) at 40 °C.

As another highly reactive organic catalyst, 1,5,7-triazabicyclo[4.4.0]dec-5-ene (TBD) was investigated next. The bicyclic guanidine base TBD has shown better efficiencies than DBU in many chemical transformations including the polymerization of lactides and cyclic carbonates [27,33]. As expected, TBD effectively promoted the polymerization of trimethylene carbonate both, in solution and under solvent-free ball-milling conditions. Nearly quantitative conversions into polymer were achieved within only 5 min (Table 4). Interestingly, TBD-based ball-milling polymerization did not allow for controlling the molecular weight distribution, resulting in a broad polydispersity (*M*_w_/*M*_n_) of 2.01 (Table 4, entry 5). As mentioned, fast initiation over chain propagation is one of the requirements in a controlled polymerization. While TBD could chemically enhance both the initiation and propagation steps, the mixing of catalyst, monomer, and initiator by heterogeneous ball milling may physically limit the initiation rate. Thus, a relatively slow initiation process resulted in poor molecular weight control. Most solution polymerizations had no issues with mixing and maintained good molecular weight control. The use of slower polymerization systems is advised for controlled polymerizations under ball-milling conditions.

**Table 4:** TBD-catalyzed polymerization of trimethylene carbonate: solution vs ball milling.^a^

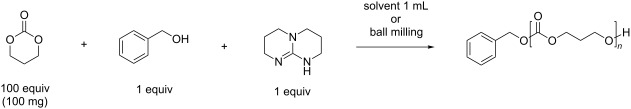						

entry	solvent	time (min)	conv (%)^b^	*M*_n_ (g/mol)^c^	*M*_w_ (g/mol)^c^	*M*_w_/*M*_n_

1	toluene	5	99	12300	20000	1.71
2	CHCl_3_	5	96	8650	9810	1.13
3	CH_2_Cl_2_	5	86	9330	10500	1.12
4	THF	5	76	7720	8750	1.09
5^d^	ball mill	5	99	12200	24400	2.01

^a^Polymerization conditions: (solution) TMC (100 mg, 100 equiv), BnOH (1.02 µL, 1 equiv), and TBD (1.4 mg, 1 equiv) in 1 mL selected solvent at rt; (ball milling) in a 10 mL stainless-steel jar with three stainless-steel balls with 7 mm diameter. ^b^Determined by ^1^H NMR spectroscopy. ^c^Determined by GPC calibrated with polystyrene standards in tetrahydrofuran (THF) at 40 °C. ^d^Average of two runs is reported.

Next, a high degree of polymerization was pursued. Polymerizations were conducted under the same conditions but with a higher monomer to initiator ratio ([TMC]:[I]:[DBU] = 200:1:2) (Table 5). The reaction reached over 90% conversion after 3 h. However, the molecular weight did not increase at all. The competitive degradation of poly(trimethyl carbonate) became significant after 100 degrees of polymerization. To validate mechanical degradation of PTMC under ball-milling conditions, high molecular weight PTMC (*M*_n_ = 22900 g/mol) was synthesized and grinded under the same mechanical conditions of Table 5, entry 1, which led to degradation to lower molecular weight (*M*_n_ = 8220 g/mol). In our previous study on poly(lactic acid) synthesis, liquid-assisted grinding (LAG), the addition of a very small amount of a liquid, prevented chain-degradation from high impact collisions, and afforded PLA with over 100,000 g/mol. Thus, LAG was also tested in the PTMC synthesis [13–14] with toluene and THF as liquids. A catalytic amount of liquid (10 or 20 µL to 100 mg TMC), however, failed to protect the poly(trimethylene carbonate) chain from mechanical degradation and similar molecular weights were obtained, regardless of LAG. The LAG for mechanochemical polymerization reactions has been studied only in limited cases so far and the exact working mechanism is currently obscure. To have a better understanding of LAG on chain protection, extensive studies are currently in progress.

**Table 5:** PTMC synthesis with a high monomer to initiator ratio.^a^

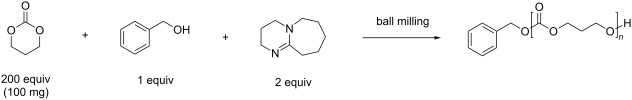						

entry	liquid additives	time	conv (%)^b^	*M*_n_ (g/mol)^c^	*M*_w_ (g/mol)^c^	*M*_w_/*M*_n_

1	none	3 h	97	10900	13600	1.24
2	toluene (10 µL)	3 h	86	11900	13100	1.10
3	toluene (20 µL)	3 h	91	11500	13700	1.19
4	THF (20 µL)	3 h	96	11400	13900	1.22

^a^Polymerization conditions: TMC (100 mg, 200 equiv), BnOH (0.49 µL, 1 equiv), and DBU (1.46 µL, 2 equiv) in a 10 mL stainless-steel jar with three 7 mm diameter stainless-steel balls. ^b^Determined by ^1^H NMR spectroscopy. ^c^Determined by GPC calibrated with polystyrene standards in tetrahydrofuran (THF) at 40 °C.

## Conclusion

A mechanochemical method, ball milling, was applied to the synthesis of poly(trimethylene carbonate). The representative organocatalyst, DBU, exhibited excellent polymerization efficiency and good chain-length control under solvent-free conditions. When compared to the very low rate obtained under solution conditions, this demonstrates that mechanochemical reactions can improve reaction efficiency and greenness. The use of TBD truly enhanced the efficiency, and all polymerizations reached completion within 5 min, despite physical mixing limitations. However, the mechanochemical polymerization was accompanied by degradation processes, which limited the molecular weight to 10,000 g/mol. Liquid-assisted grinding did not show any protective effect, and the search for other parameters to mitigate polymer-chain breaking is currently in progress.

## Experimental

**General considerations.** Chemical reagents obtained from commercial sources were used without further purification. 1,8-Diazabicyclo[5.4.0]undec-7-ene (DBU) was distilled over CaH_2_. All solvents (THF, CH_2_Cl_2_, CHCl_3_, and toluene) were dried over a mixture of pre-activated neutral alumina and 3 Å molecular sieves. A Retsch Mixer Mill MM 400 was used for the ball-milling experiments with a 10 mL stainless-steel vessel and 7 mm stainless balls. ^1^H NMR spectra were recorded with a 400 MHz Bruker Avance III HD Fourier transform NMR spectrometer and all signals were referenced to residual protonated solvent. Gel permeation chromatography (GPC) analyses with refractive index (RI) detection were used to determine the number-averaged molecular weights (*M*_n_), weight-averaged molecular weights (*M*_w_), and polydispersities (*M*_w_/*M*_n_). The RI measurements were carried out using an instrument set composed of a Waters 1515 isocratic pump, a 2414 differential refractive index detector, and a column-heating module with Shodex KF-804, KF-803, and KF-802.5 columns in series. The columns were eluted with tetrahydrofuran (preservative-free HPLC grade, Fisher) at 40 °C at 1.0 mL/min and calibrated using 14 monodisperse polystyrene standards (Alfa Aesar). The temperature was recorded using a Fluke VT04 Visual IR thermometer.

**Synthesis of trimethylene carbonate.** 1,3-Propanediol (4.72 mL, 0.657 mol) and ethyl chloroformate (12.5 mL, 0.131 mol) were dissolved in anhydrous THF (0.13 L). The mixture was stirred in an ice bath for 1 h and a solution of triethylamine (19.2 mL, 0.138 mol) in THF (9 mL) was slowly added. Then, the solution was transferred to ambient temperature and stirred for 2 h. The reaction mixture was filtered and the volume of the solution was reduced to 40–50 mL. The mixture was kept in a freezer for 12 h and the precipitate was recovered by filtration. The recovered solid was recrystallized in ethyl acetate and sublimed (2.9 g, 43%). ^1^H NMR (400 MHz, CDCl_3_) δ 4.47–4.44 (t, 4H), 2.18–2.12 (quintet, 2H).

**Representative procedure for mechanochemical solvent-free poly(trimethylene carbonate) synthesis** (Table 1, entry 11). Three 7 mm stainless-steel milling balls were placed in a 10 mL stainless-steel milling container and trimethylene carbonate (0.100 g), benzyl alcohol (1.02 µL), and DBU (1.46 µL) were added. The milling vessel was placed in a vibrational ball mill and vibrated at 30 Hz. After 2 hours, the vessel was opened and benzoic acid (10 mg) was added followed by an additional 5 minutes of milling to quench the polymerization. To avoid data inconsistency due to inhomogeneity, all material was dissolved in methylene chloride and an aliquot was subjected to analysis by ^1^H NMR spectroscopy and GPC measurements to determine the conversion and molecular weight.

## Supporting Information

File 1 Raw data for tables, GPC and NMR spectra.
