# Supplementary material for: Mechanochemical synthesis of poly(trimethylene carbonate)s: an example of rate acceleration
Source: Beilstein J Org Chem. 2019 Apr 23;15:963–70. doi: 10.3762/bjoc.15.93 (PMC6541340; doi:10.3762/bjoc.15.93)

BEILSTEIN JOURNAL OF ORGANIC CHEMISTRY

Supporting Information

for

Mechanochemical synthesis of poly(trimethylene carbonate)s: an example of rate acceleration

Sora Park and Jeung Gon Kim

Beilstein J. Org. Chem. **2019,** *15,* 963–970. doi:10.3762/bjoc.15.93

Raw data for tables, GPC and NMR spectra

License and Terms: This is a supporting information file under the terms of the Creative Commons Attribution License (http://creativecommons.org/licenses/by/4.0). Please note that the reuse, redistribution and reproduction in particular requires that the authors and source are credited.
The license is subject to the *Beilstein Journal of Organic Chemistry* terms and conditions: (https://www.beilstein-journals.org/bjoc)

Table of contents

A. Raw data for tables S2

B. High molecular weight PTMC synthesis and its degradation S7

C. Example of NMR peak assignment (Table S1, entry 10-1) S8

D. GPC and NMR Spectra S9

A. Raw data for tables

Table S1: DBU-catalyzed polymerization of trimethylene carbonate: solution vs. ball-milling.

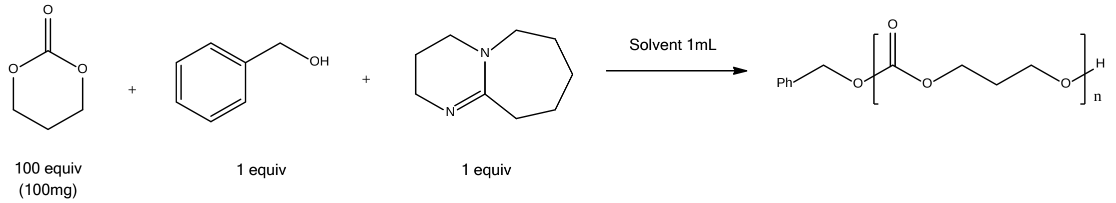

Entry	Solvnet	Time(h)	Conv (%)[b]	M_n (kg/mol)[c]	M_w (kg/mol)[c]	M_w/M_n [c]	Condition
1	Chloroform	1	1	-	-	-	solution,
2		24	24	2120	2270	1.07	
3	THF	1	<1	-	-	-	solution
4		24	5	-	-	-	
5	Toluene	1	<1	-	-	-	solution
6		24	23	2660	2950	1.11	
7	CH_2Cl_2	1	3	-	-	-	solution
8		24	43	3990	4200	1.05	
9-1	Ball Mill No-solvent	0.5	44	3990	4370	1.09	10mL container 7mm ball * 3 30Hz
9-2		0.5	41	3870	4330	1.12	
10-1		1	70	7130	8020	1.12	
10-2		1	80	7620	8680	1.14	
11-1		2	91	9420	11020	1.17	
11-2		2	95	9030	10130	1.12	

Table S2: Vibration and ball size effects.

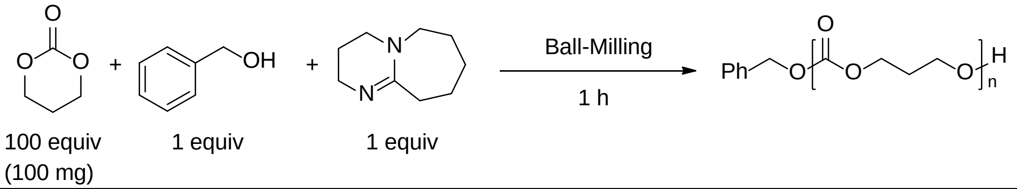

entry	Frequency and Ball diameter	Conv (%)[c]	M_n (g/mol)[d]	M_w (g/mol)[d]	M_w/M_n
1-1	10 Hz, 7 mm x 3 ea	59	5710	6340	1.11
1-2		60	5680	6290	1.11
2-1	20 Hz, 7 mm x 3 ea	65	5980	6340	1.09
2-2		61	6030	6680	1.11
3-1	30 Hz, 7 mm x 3 ea	70	7130	8020	1.12
3-2		80	7620	8680	1.14
4-1	30 Hz, 7 mm x 5 ea	89	7120	8150	1.14
4-2		87	6640	7470	1.13
5-1	30 Hz, 12 mm x 1 ea	81	6610	7420	1.12
5-2		86	6710	7590	1.13

* Entries 3-1 and 3-2 are from entries 10-1 and 10-2 of Table S1.

Table S3. DBU-catalyzed polymerization of trimethylene carbonate at 40 °C.

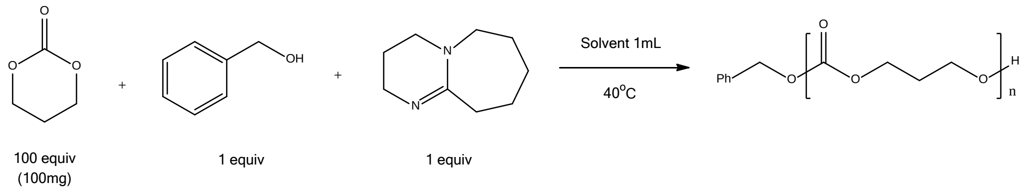

Entry	Solvnet	Time(h)	Conv (%)[b]	M_n (kg/mol)[c]	M_w (kg/mol)[c]	M_w/M_n [c]	Condition
1	Chloroform	1	3	-	-	-	solution, 40℃
2		24	41	3490	3770	1.08	
3	THF	1	<1	-	-	-	solution, 40℃
4		24	6	-	-	-	
5	Toluene	1	<1	-	-	-	solution, 40℃
6		24	70	7130	8380	1.17	
7	CH_2Cl_2	1	4	-	-	-	solution, 40℃
8		24	41	3000	3210	1.07	
9-1	Ball Mill No-solvent	1	70	7130	8020	1.12	
9-2		1	80	7620	8680	1.14	
10-1	Ball Mill No-solvent	2	91	9420	11020	1.17	Final temperature 36.3 °C
10-2		2	95	9030	10130	1.12	Final temperature 36.1 °C

* Entries 9 and 10 are from entries 10 and 11 of Table S1.

Table S4. TBD catalyzed polymerization of trimethylene carbonate: solution vs. ball-milling.

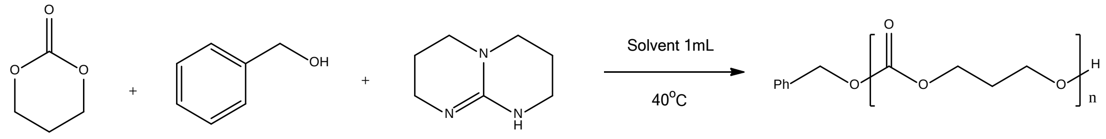

Entry	Solvnet	Time(min)	Conv (%)[b]	M_n (kg/mol)[c]	M_w (kg/mol)[c]	M_w/M_n [c]	Condition
1	Toluene	5	99	12300	19970	1.62	Solution
2	Chloroform	5	96	8650	9810	1.13	Solution
3	CH_2Cl_2	5	86	9330	10530	1.13	Solution
4	THF	5	76	7710	8750	1.13	solution
5-1	Ball-Mill	5	98	13250	28810	2.17	10mL container 7mm ball * 3 30Hz
5-2		5	99	11120	20050	1.80	

Table S5. PTMC synthesis with a high monomer to initiator ratio.

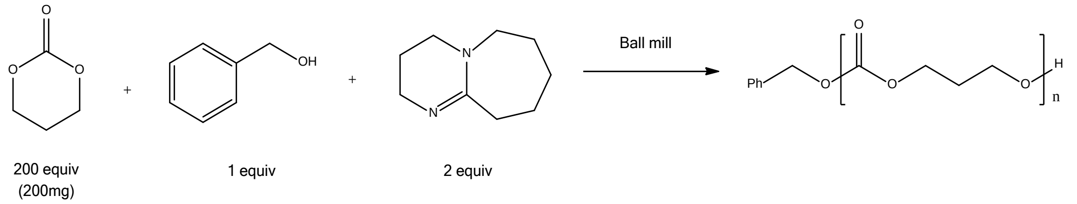

Entry	Solvnet	Time(h)	Conv (%)[b]	M_n (kg/mol)[c]	M_w (kg/mol)[c]	M_w/M_n [c]	Condition
1-1	Ball mill (No solvent)	3	97	10800	13690	1.27	10mL container 7mm ball * 3 30Hz
1-2		3	98	11010	13510	1.23	
2	Ball mill (Toluene 10μL)	3	86	11940	13090	1.09	10mL container 7mm ball * 3 30Hz
3	Ball mill (Toluene 20μL)	3	93	11520	13690	1.19	10mL container 7mm ball * 3 30Hz
4	Ball mill (THF 20μL)	3	96	11400	13940	1.22	10mL container 7mm ball * 3 30Hz

B. High molecular weight PTMC synthesis and its degradation

Experimental procedure

To the solution of trimethylene carbonate (0.25 g) and benzyl alcohol (0.50 μL) in $CHCl_3$ (0.7 mL), TBD (3.3 mg) in $CHCl_3$ (0.5 mL) were added. After 30 min, benzoic acid (10 mg) was added to quench the polymerization reaction. The resulting solution was poured into cold methanol (15 mL) to precipitate PTMC. The precipitation were repeated twice. The viscous PTMC was dried under vacuum (190 mg, M_n = 22900 g/mol, M_w = 33900 g/mol).

Three 7 mm stainless-steel milling balls were placed in a 10 mL stainless-steel milling container and PTMC (0.10 g) was added. The milling vessel was placed in a vibrational ball mill and vibrated (30 Hz). After 2 hours, the vessel was opened and an aliquot was taken for the GPC measurement. (M_n = 8220 g/mol, M_w = 16300 g/mol).

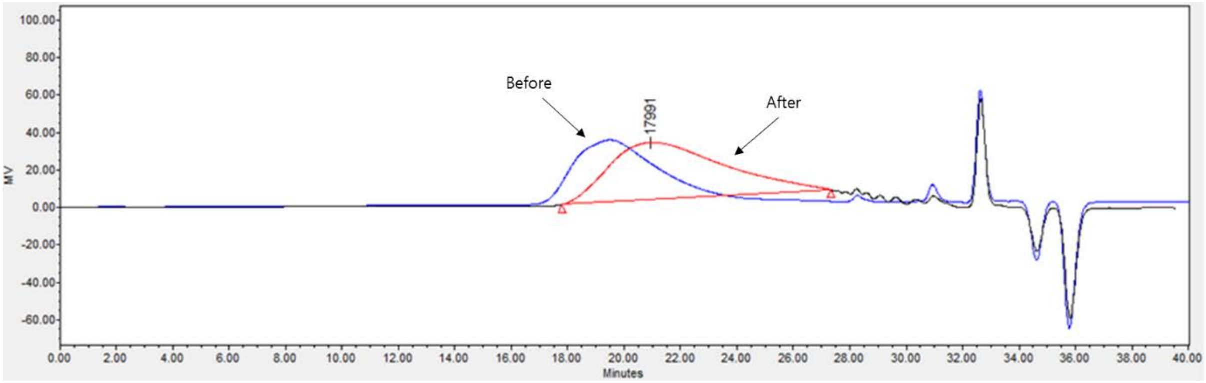

C. Example of NMR peak assignment (Table S1, entry 10-1)

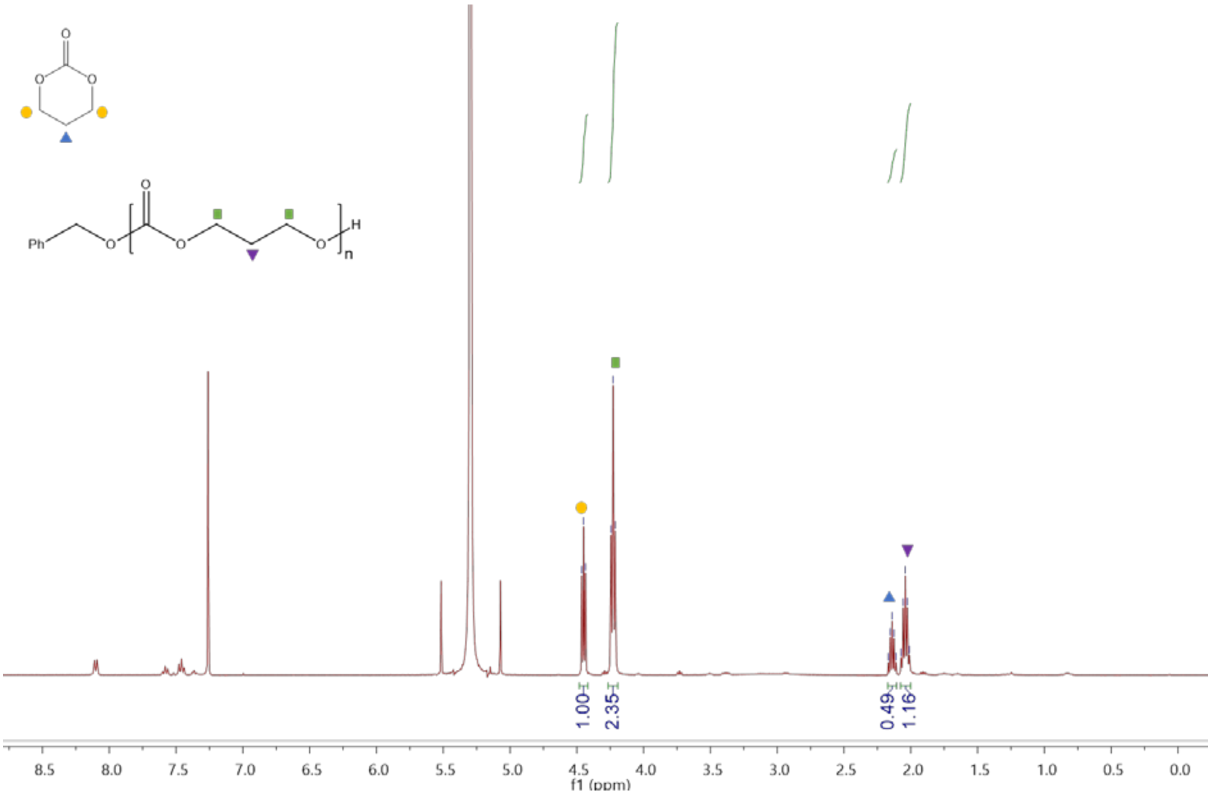

D. GPC and NMR Spectra of Table 1-4

Table 1, entry 1

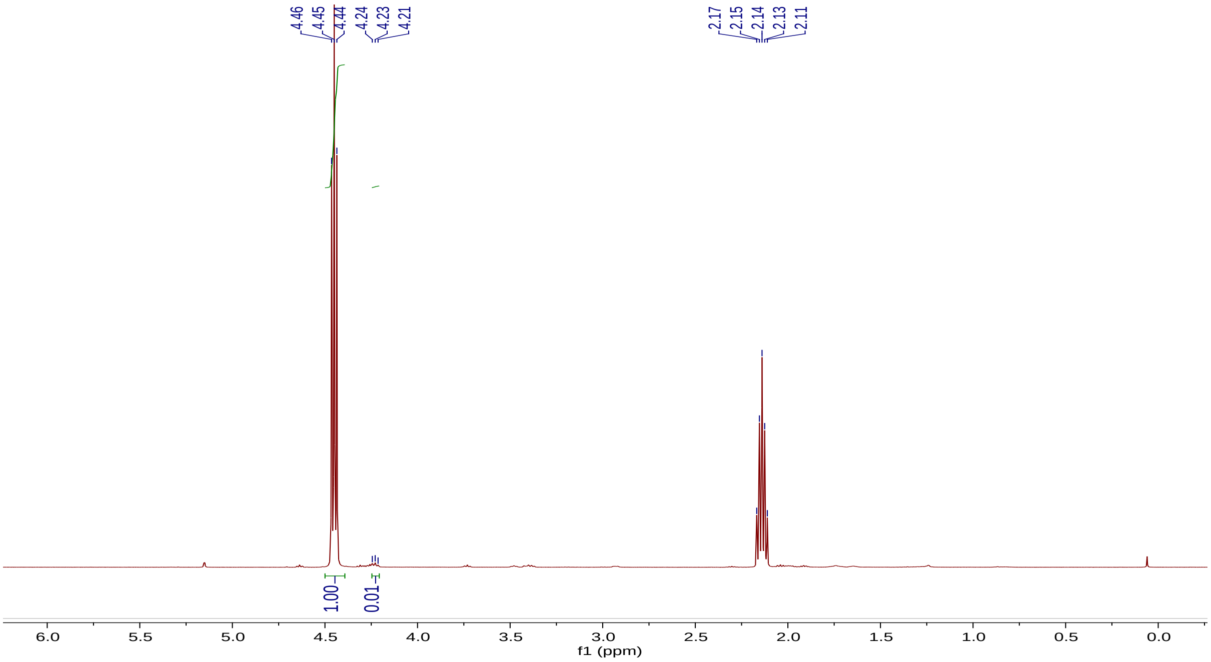

Table 1, entry 2

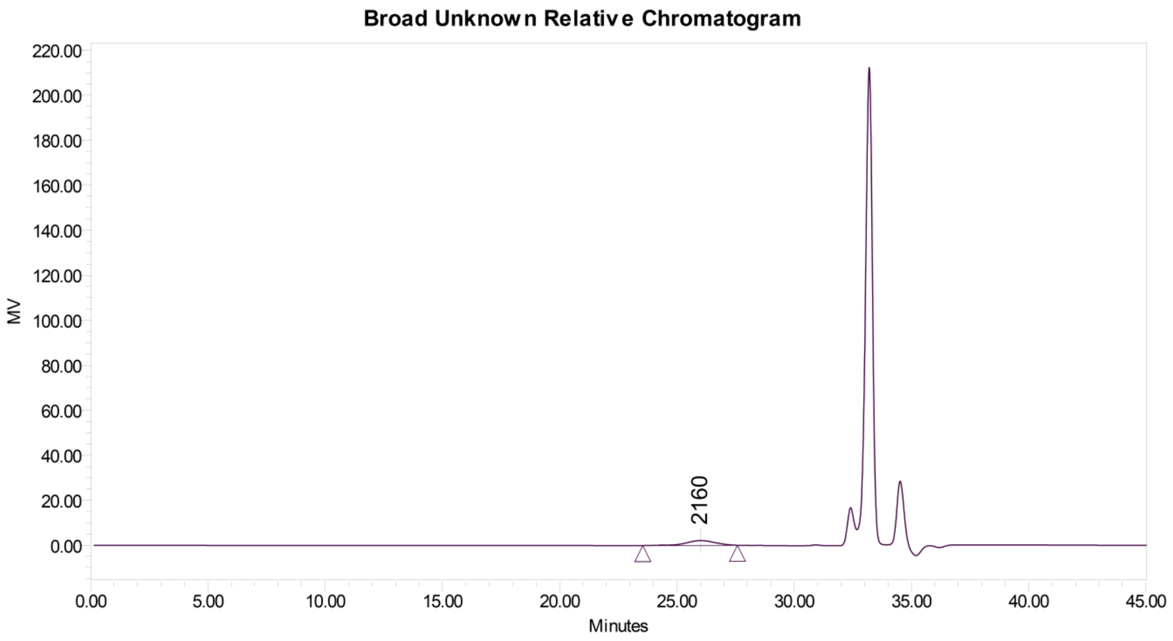

Broad Unknown Relative Peak Table

	Distribution Name	Mn (Daltons)	Mw (Daltons)	MP (Daltons)	Mz (Daltons)	Mz+1 (Daltons)	Polydispersity	Mz/Mw	Mz+1/Mw
1		2122	2272	2160	2460	2700	1.070627	1.082662	1.188469

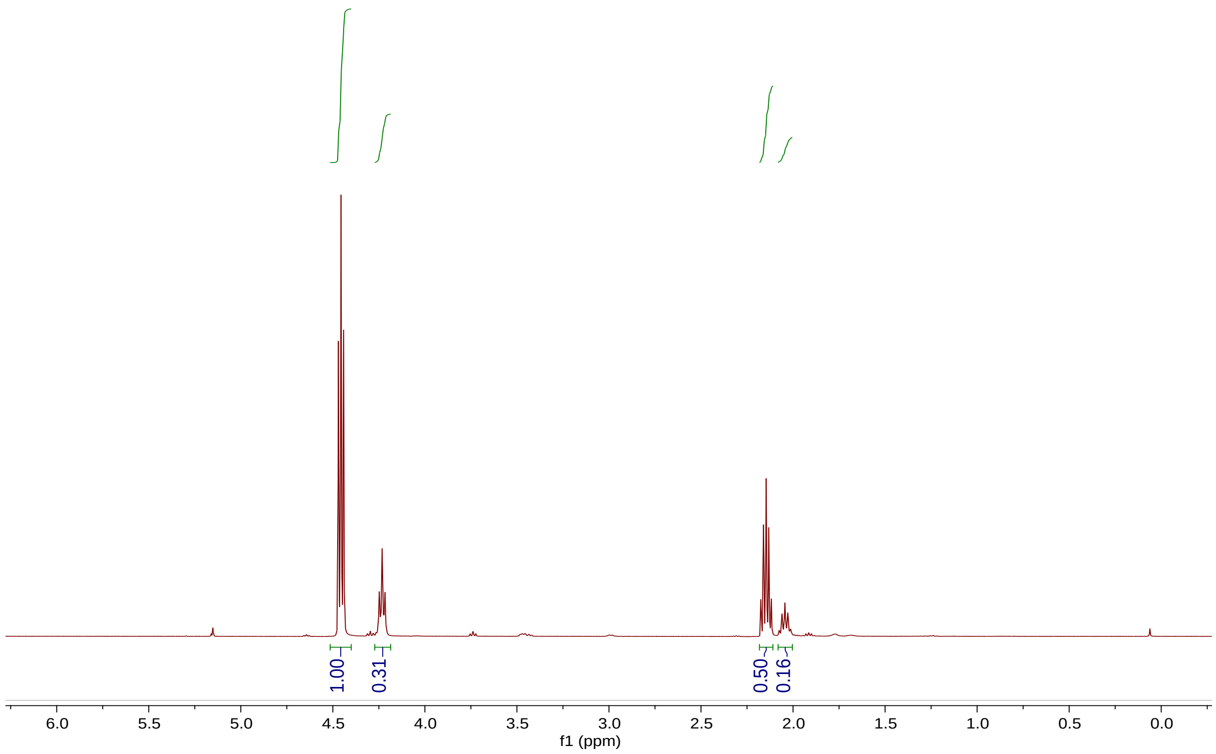

Table 1, entry 3

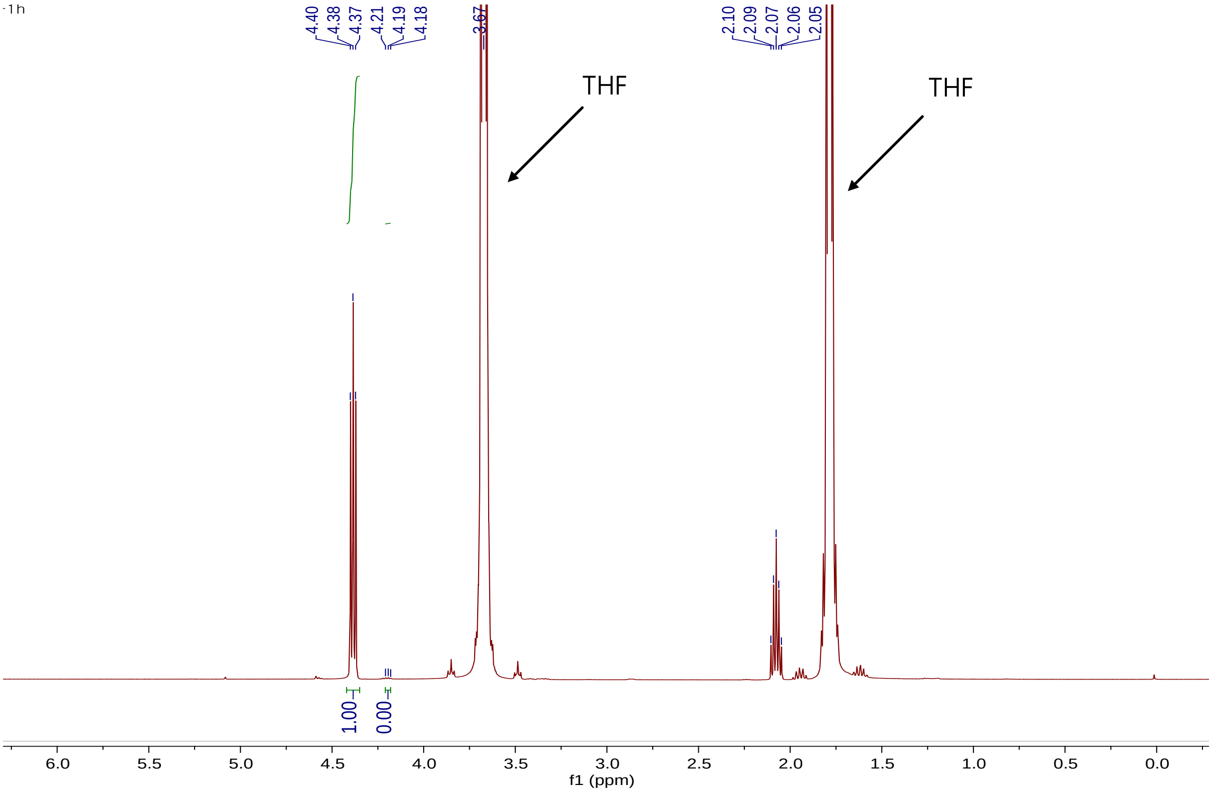

Table 1, entry 4

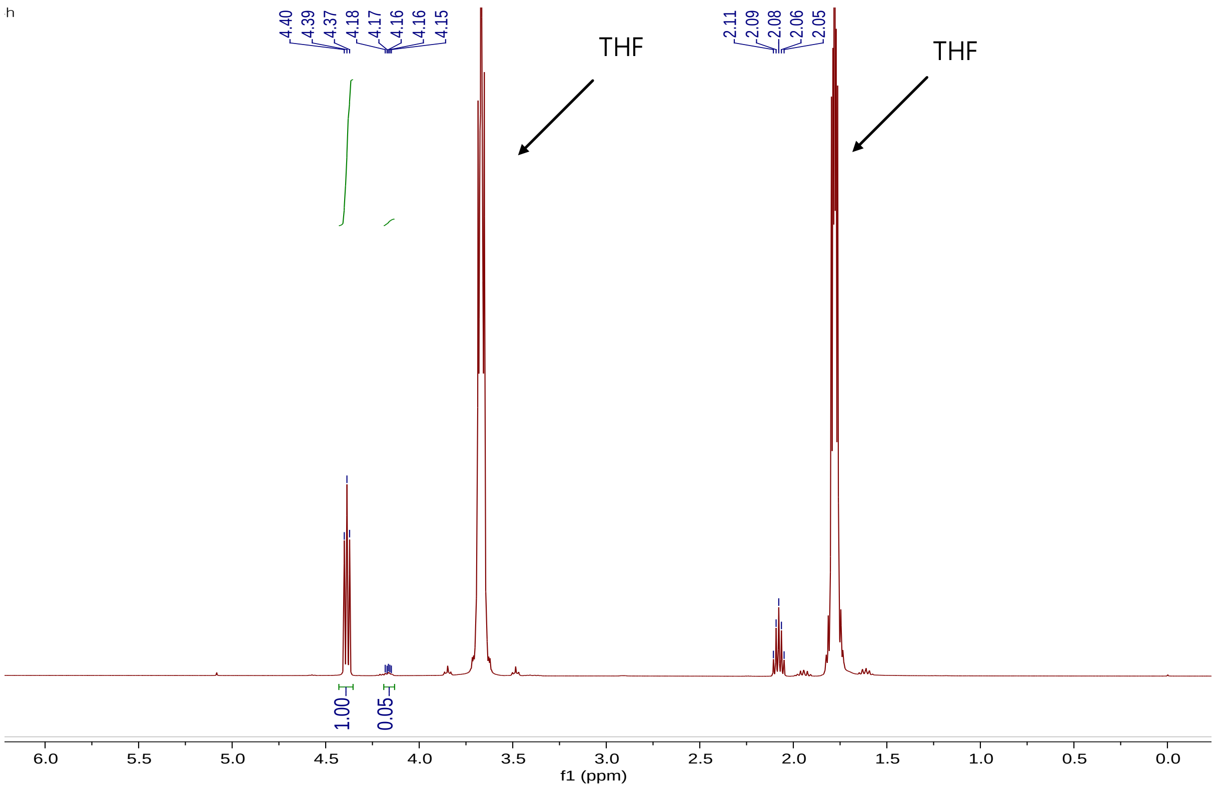

Table 1, entry 5

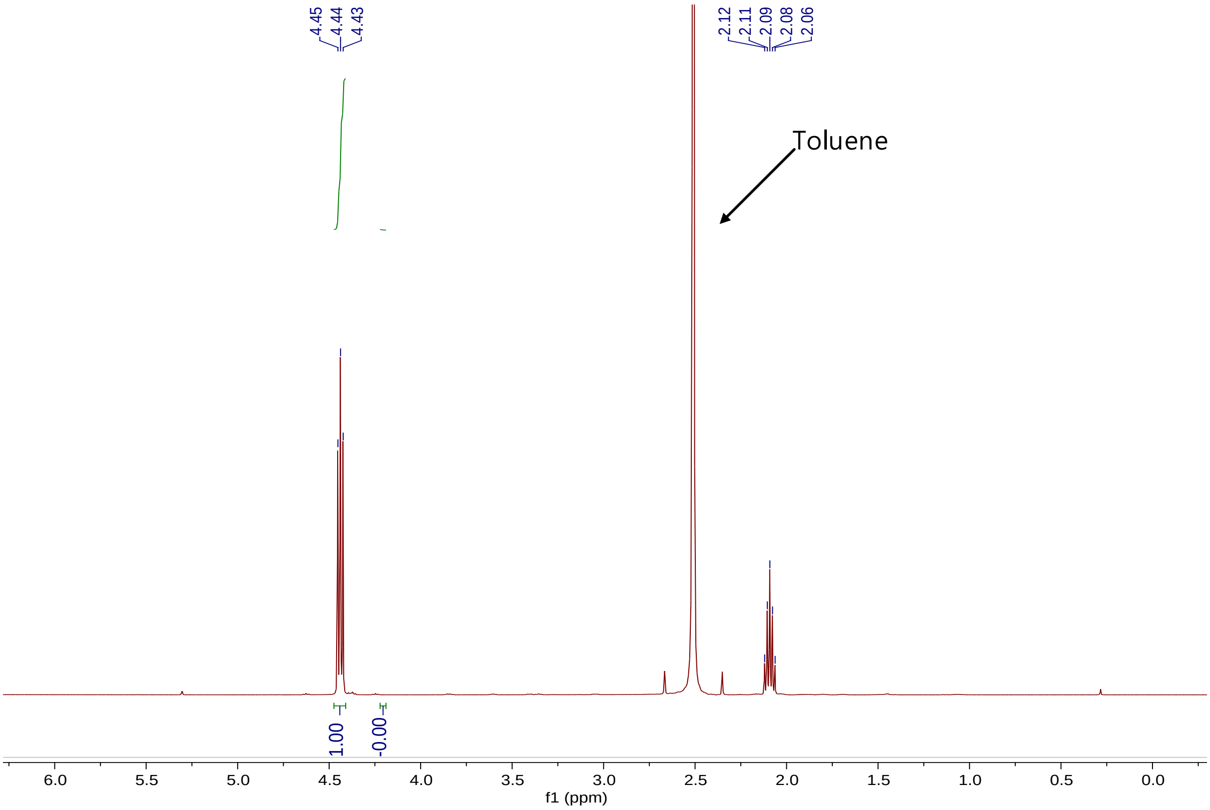

Table 1, entry 6

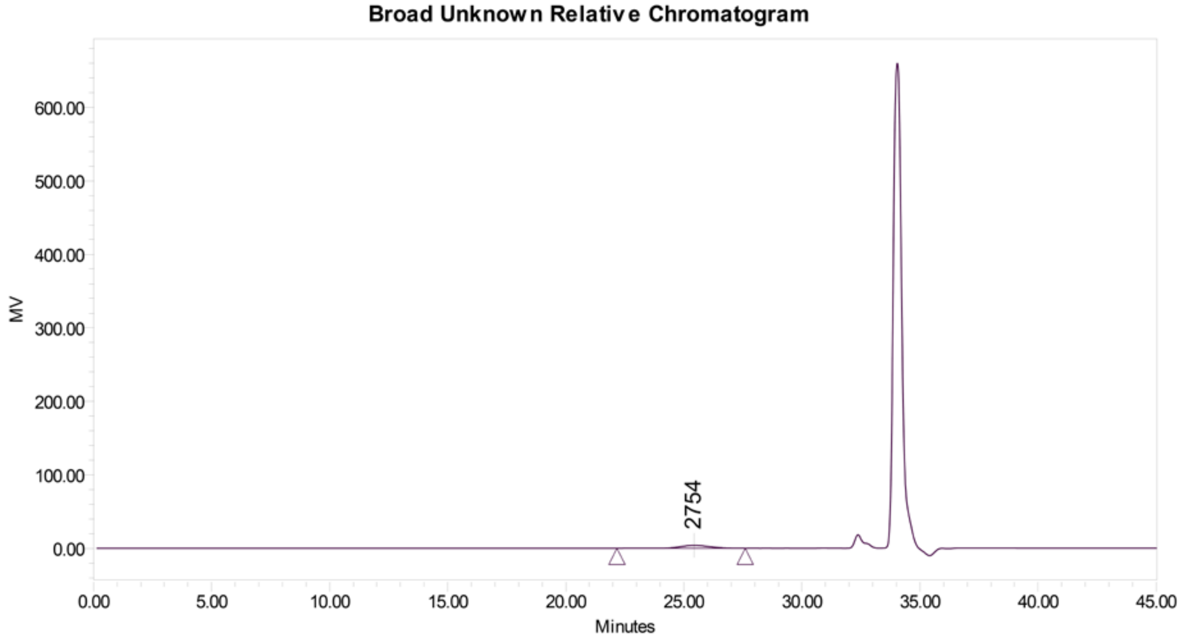

Broad Unknown Relative Peak Table

	Distribution Name	Mn (Daltons)	Mw (Daltons)	MP (Daltons)	Mz (Daltons)	Mz+1 (Daltons)	Polydispersity	Mz/Mw	Mz+1/Mw
1		2660	2946	2754	3430	4281	1.107876	1.164053	1.452897

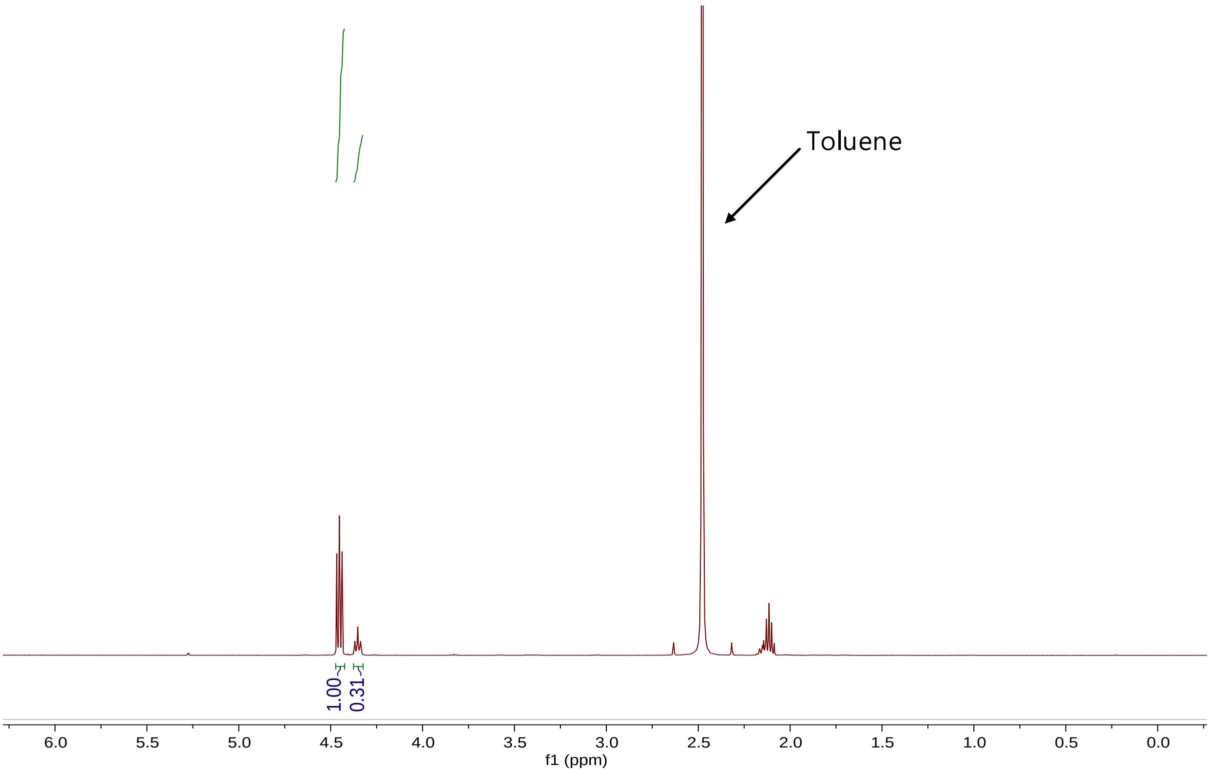

Table 1, entry 7

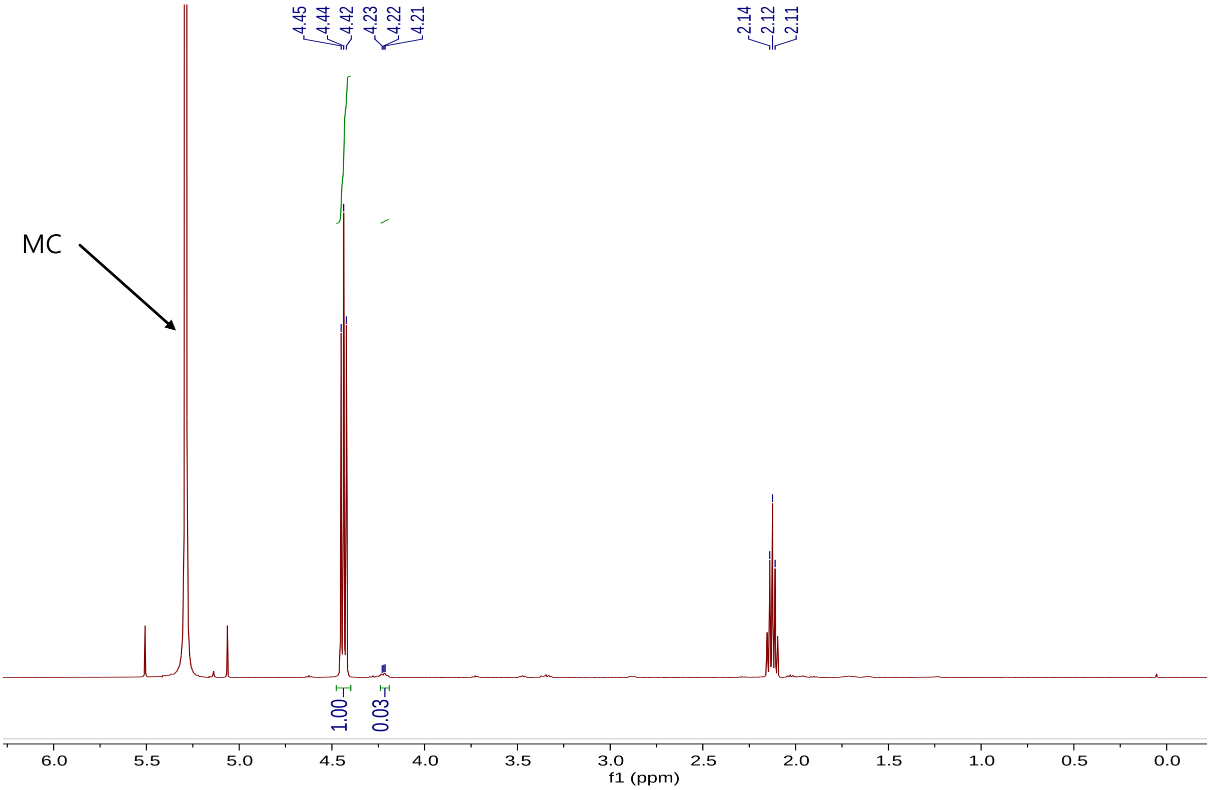

Table 1, entry 8

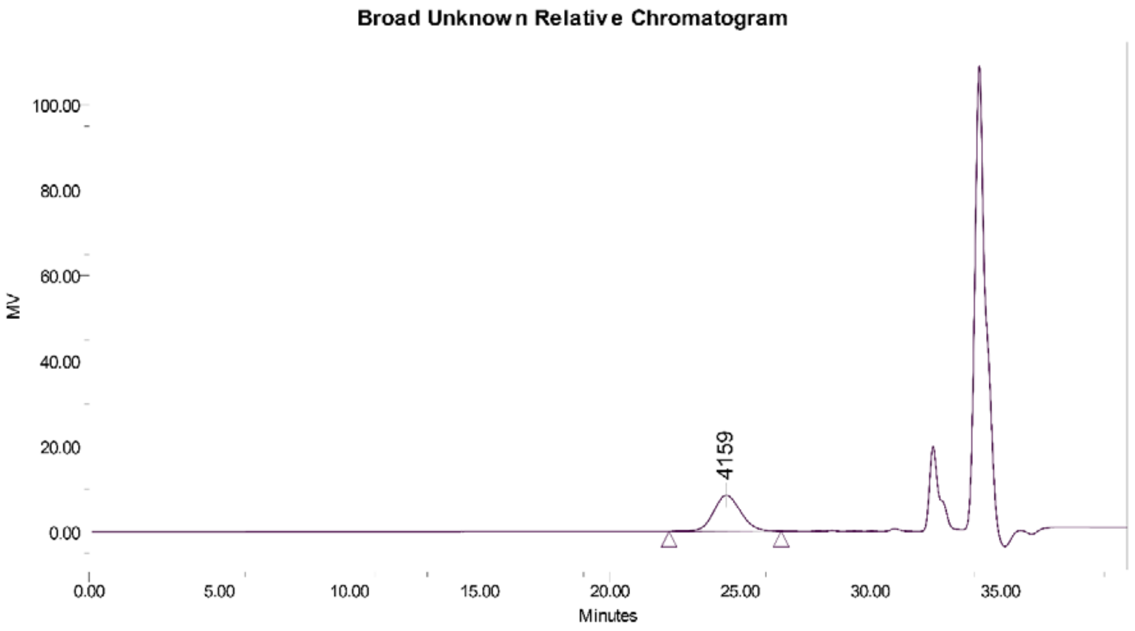

Broad Unknown Relative Peak Table

	Distribution Name	Mn (Daltons)	Mw (Daltons)	MP (Daltons)	Mz (Daltons)	Mz+1 (Daltons)	Polydispersity	Mz/Mw	Mz+1/Mw
1		3986	4196	4159	4429	4698	1.052831	1.055417	1.11966[illegible]

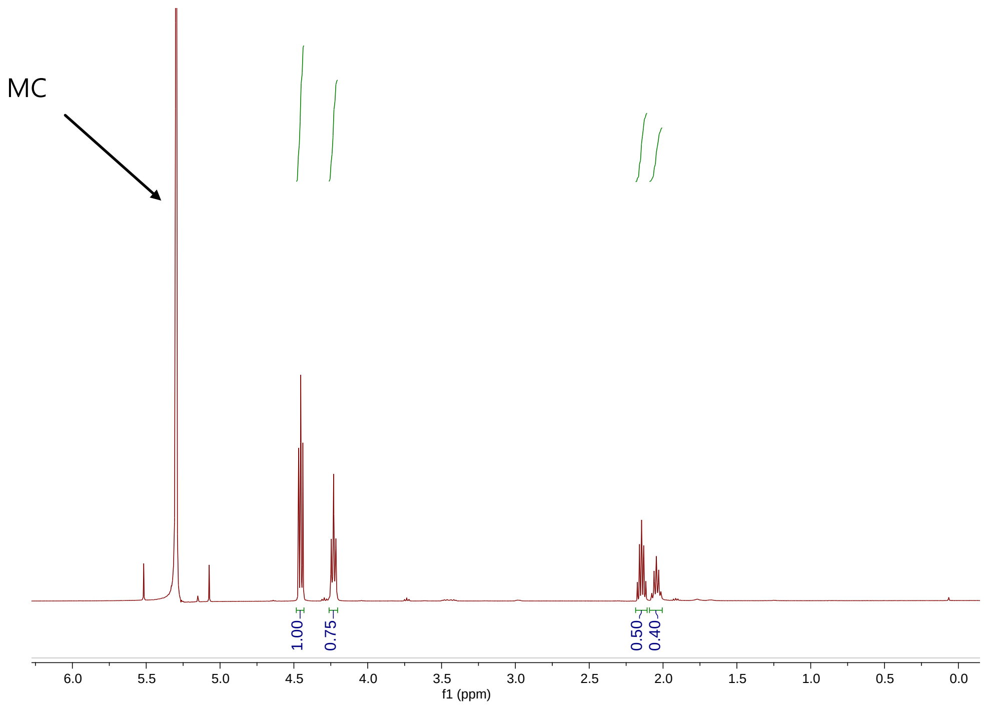

Table 1, entry 9 (Table S1, entry 9-1)

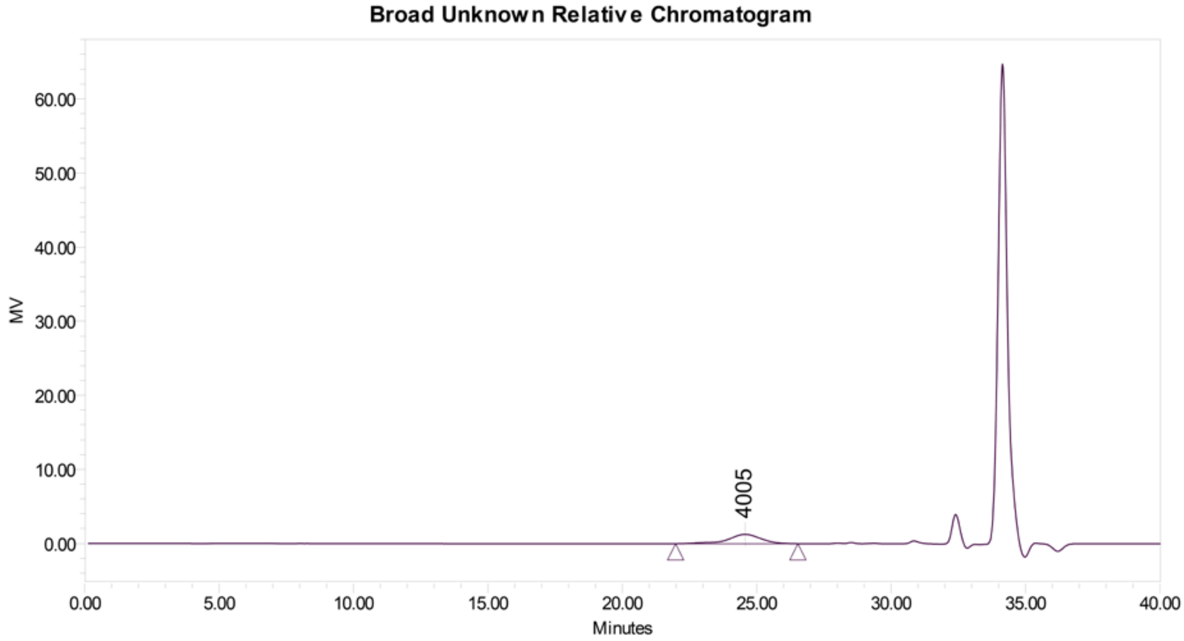

Broad Unknown Relative Peak Table

	Distribution Name	Mn (Daltons)	Mw (Daltons)	MP (Daltons)	Mz (Daltons)	Mz+1 (Daltons)	Polydispersity	Mz/Mw	Mz+1/Mw
1		3995	4372	4005	4866	5501	1.094458	1.112977	1.258153

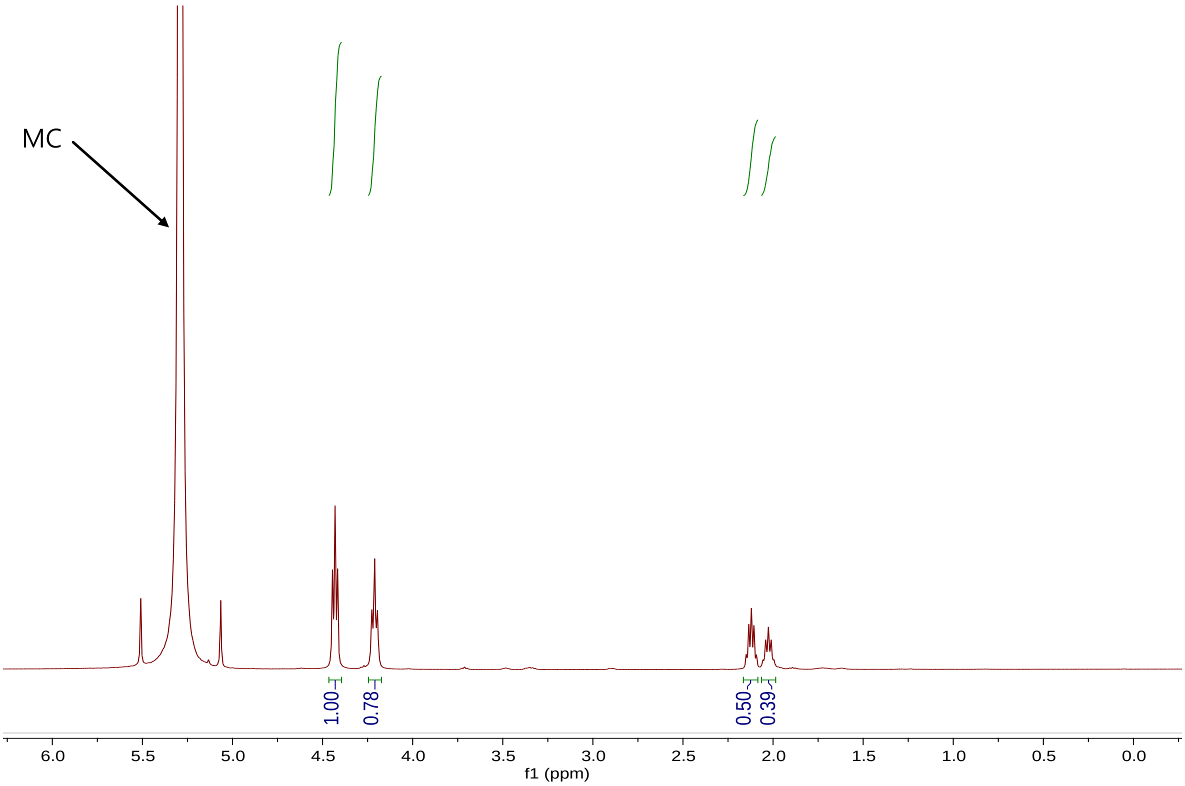

Table 1, entry 10 (Table S1, entry 10-1)

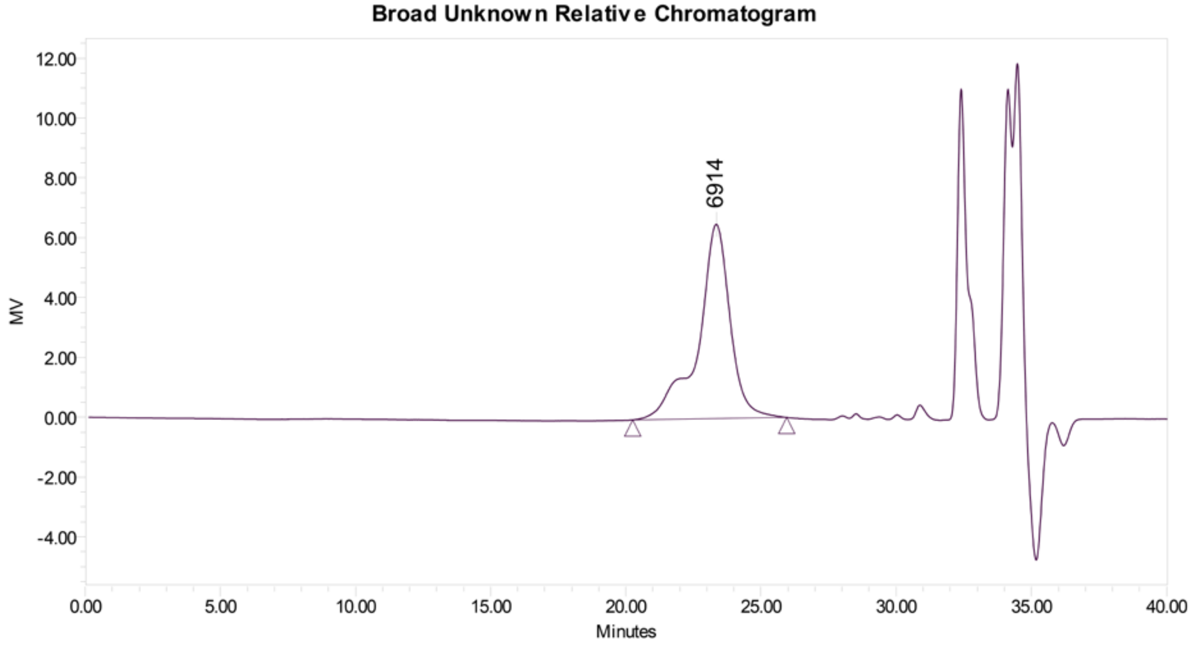

Broad Unknown Relative Peak Table

	Distribution Name	Mn (Daltons)	Mw (Daltons)	MP (Daltons)	Mz (Daltons)	Mz+1 (Daltons)	Polydispersity	Mz/Mw	Mz+1/Mw
1		7135	8018	6914	9201	10724	1.123805	1.147593	1.337472

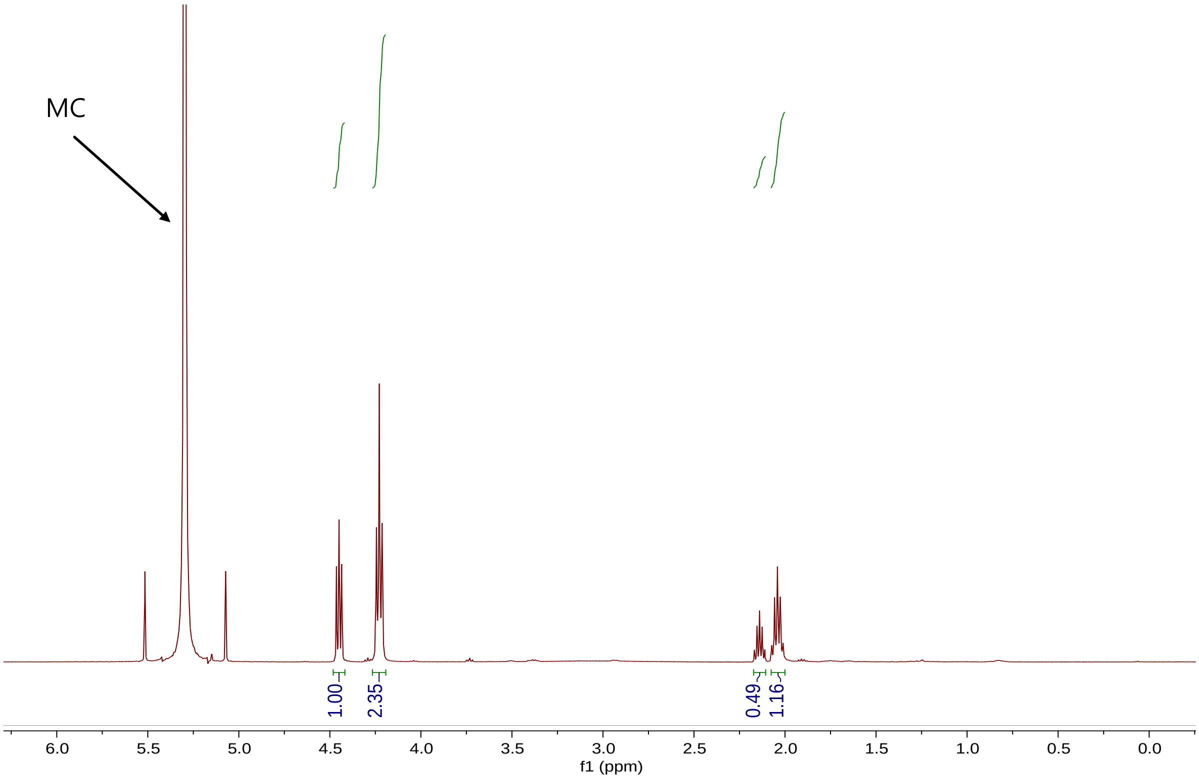

Table 1, entry 11 (Table S1, entry 11-1)

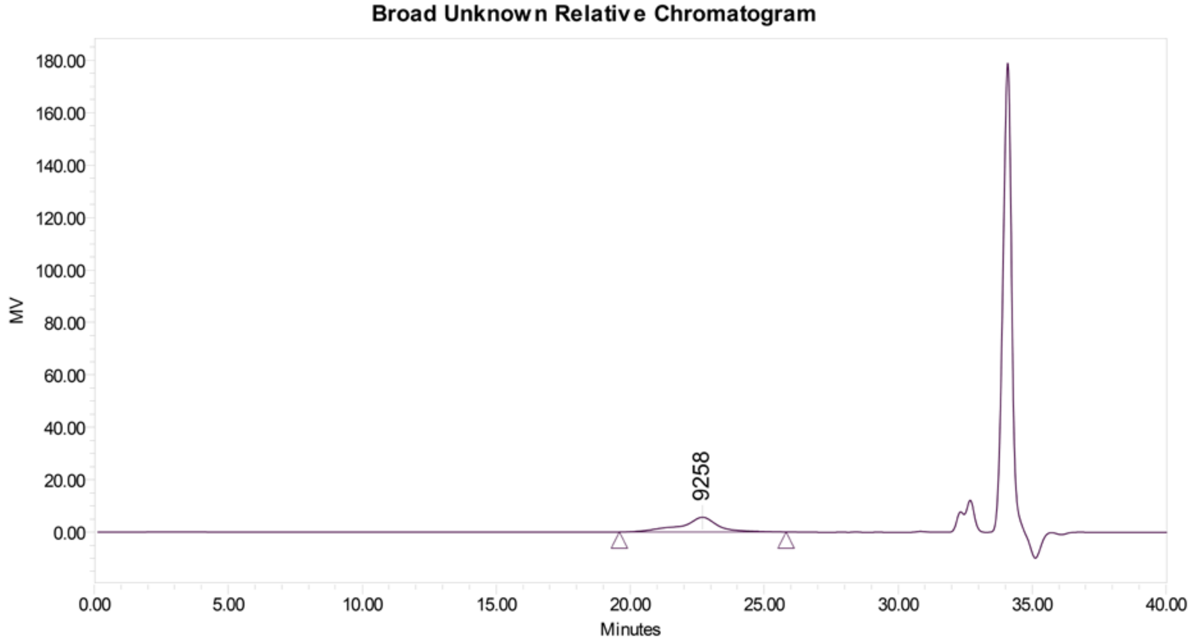

Broad Unknown Relative Peak Table

	Distribution Name	Mn (Daltons)	Mw (Daltons)	MP (Daltons)	Mz (Daltons)	Mz+1 (Daltons)	Polydispersity	Mz/Mw	Mz+1/Mw
1		9419	11024	9258	12957	15226	1.170403	1.175296	1.38113C

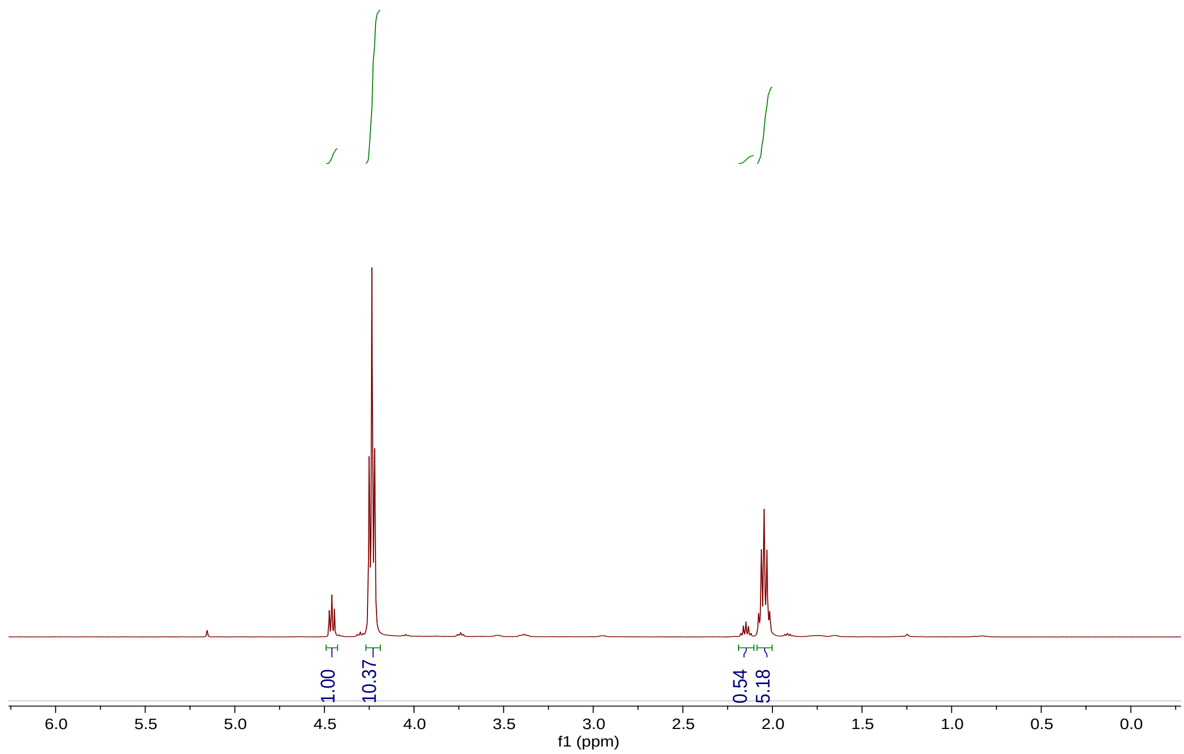

Table 2, entry 1 (Table S2, entry 1-1)

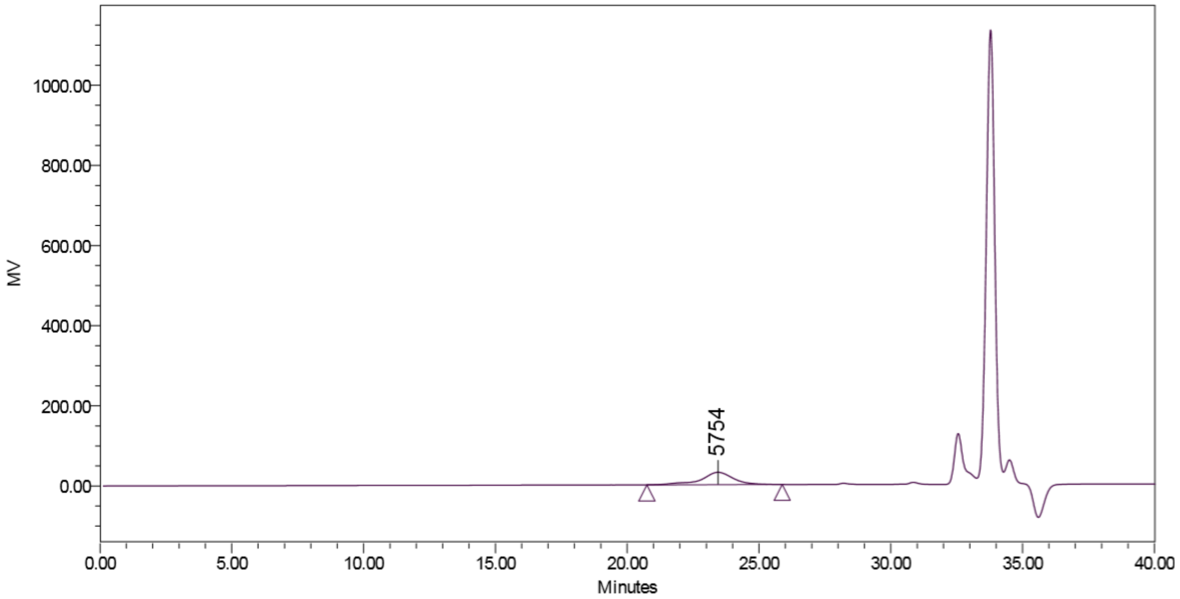

Broad Unknown Relative Peak Table

	Distribution Name	Mn (Daltons)	Mw (Daltons)	MP (Daltons)	Mz (Daltons)	Mz+1 (Daltons)	Polydispersity	Mz/Mw	Mz+1/Mw
1		5711	6342	5754	7159	8204	1.110534	1.128841	1.293657

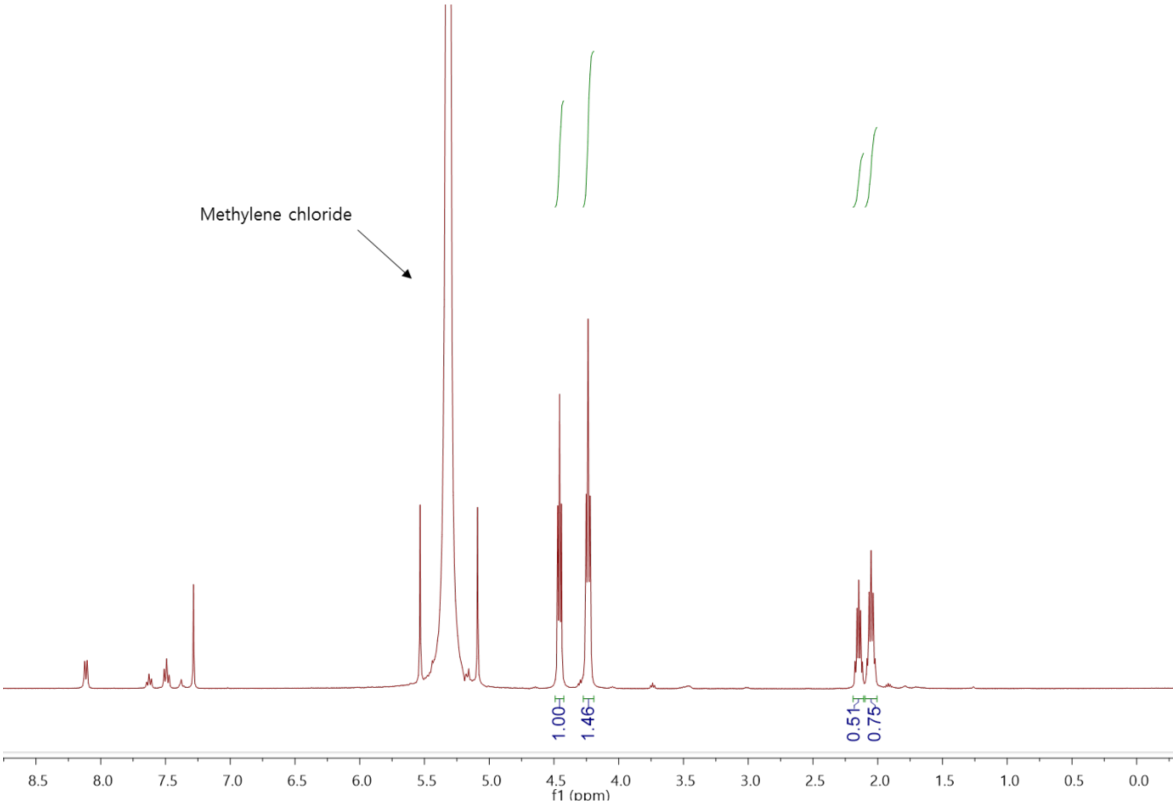

Table 2, entry 2 (Table S2, entry 2-1)

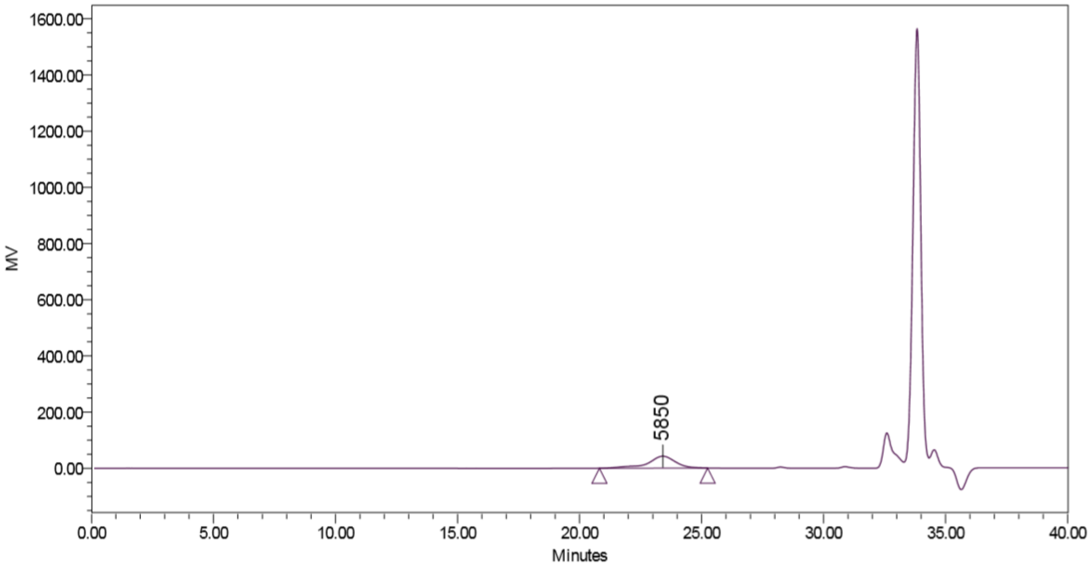

Broad Unknown Relative Peak Table

	Distribution Name	Mn (Daltons)	Mw (Daltons)	MP (Daltons)	Mz (Daltons)	Mz+1 (Daltons)	Polydispersity	Mz/Mw	Mz+1/Mw
1		5978	6537	5850	7289	8270	1.093572	1.115043	1.265050

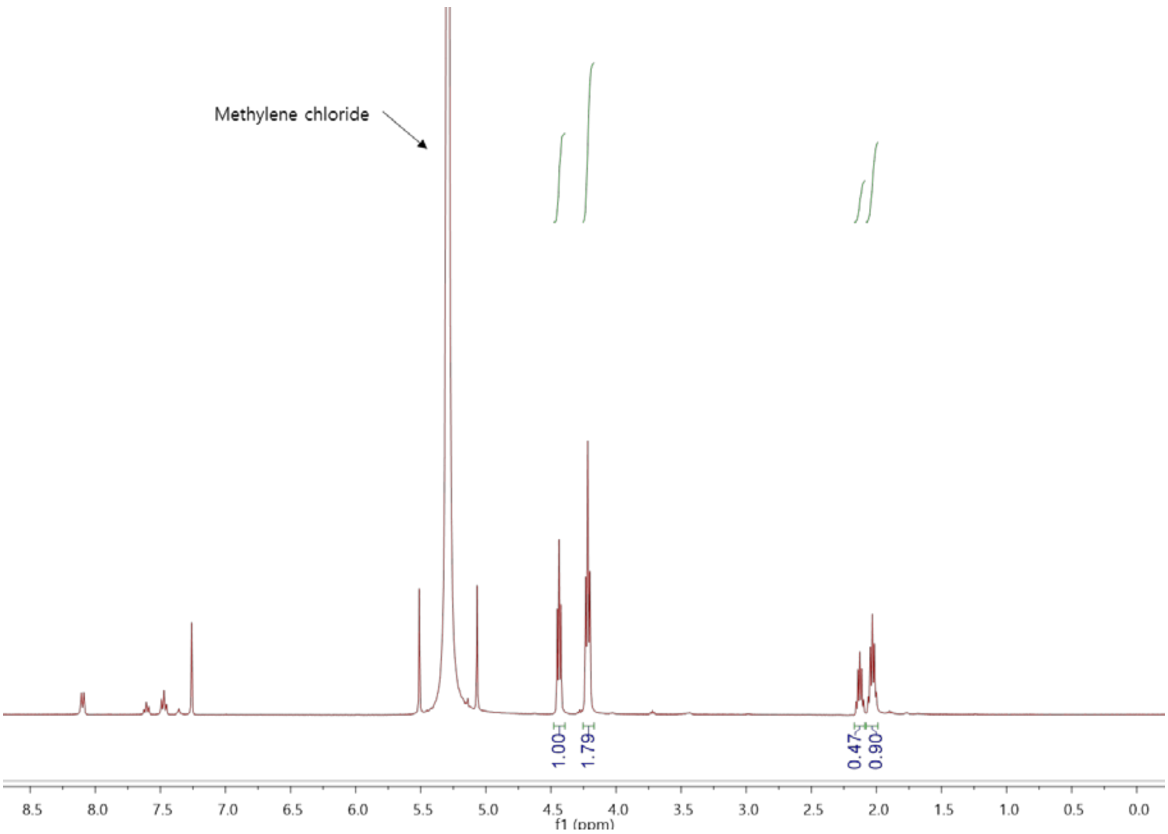

Table 2, entry 4 (Table S2, entry 4-1)

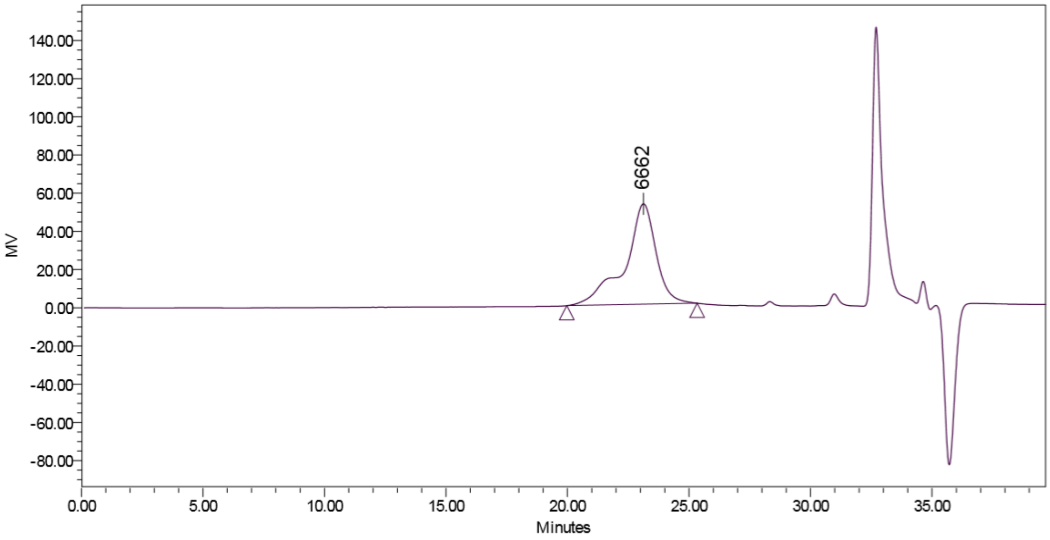

Broad Unknown Relative Peak Table

	Distribution Name	Mn (Daltons)	Mw (Daltons)	MP (Daltons)	Mz (Daltons)	Mz+1 (Daltons)	Polydispersity	Mz/Mw	Mz+1/Mw
1		7120	8152	6662	9586	11401	1.144941	1.175849	1.398497

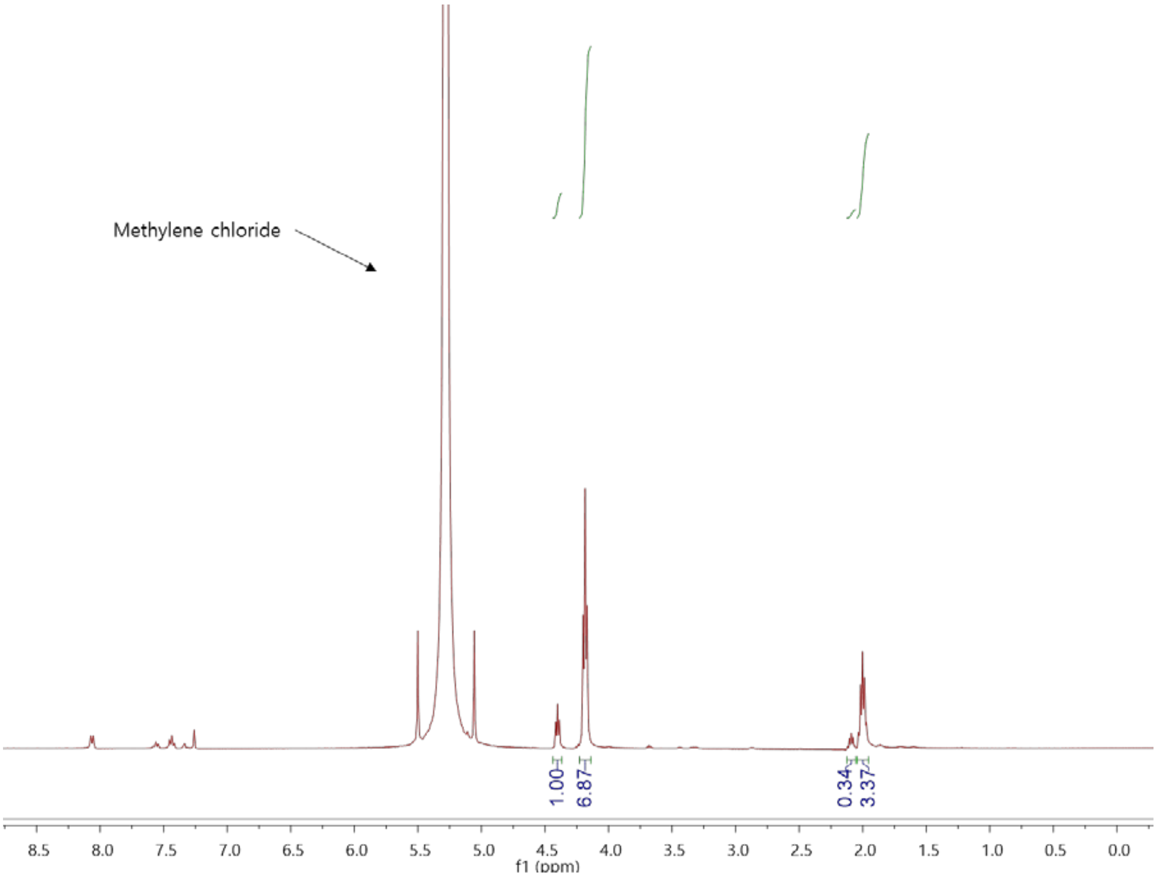

Table 2, entry 5 (Table S2, entry 5-1)

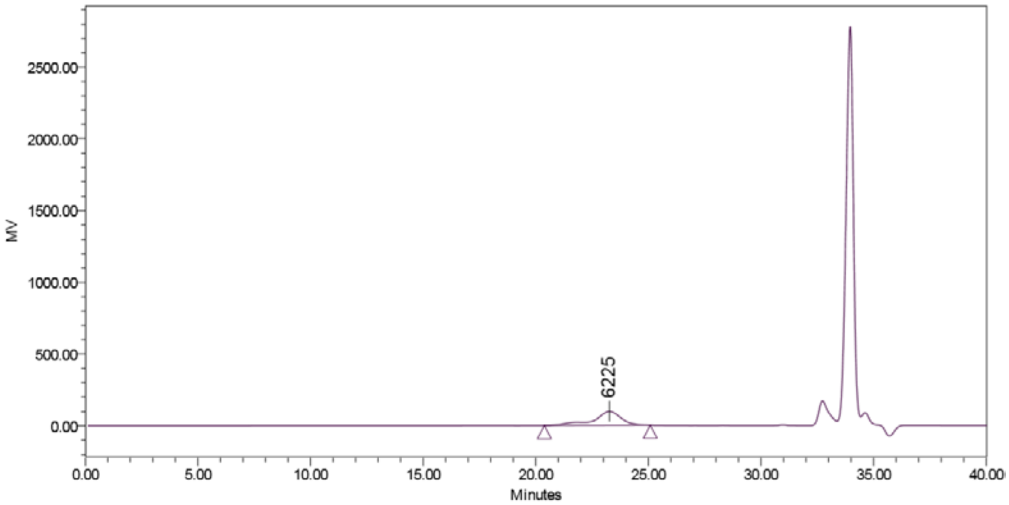

Broad Unknown Relative Peak Table

	Distribution Name	Mn (Daltons)	Mw (Daltons)	MP (Daltons)	Mz (Daltons)	Mz+1 (Daltons)	Polydispersity	Mz/Mw	Mz+1/Mw
1		6614	7420	6225	8562	10033	1.121987	1.153844	1.352092

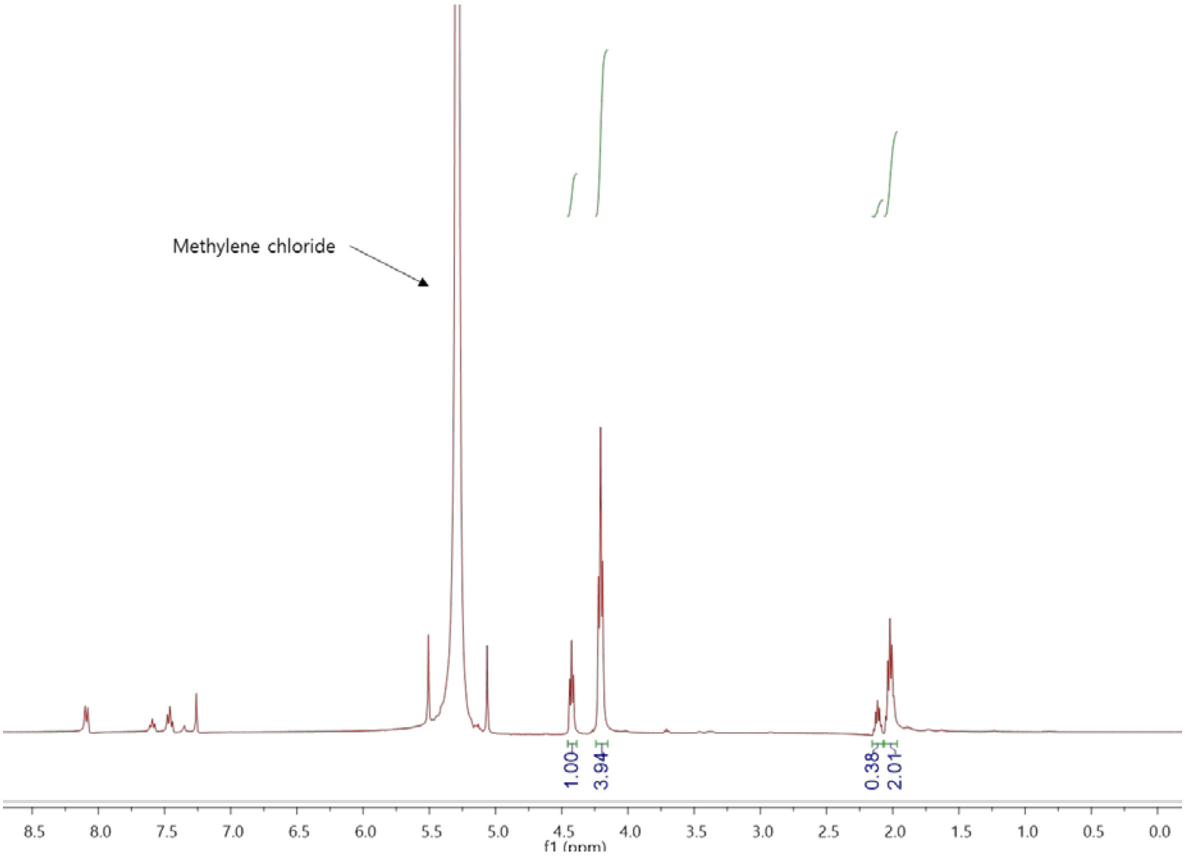

Table 3, entry 1

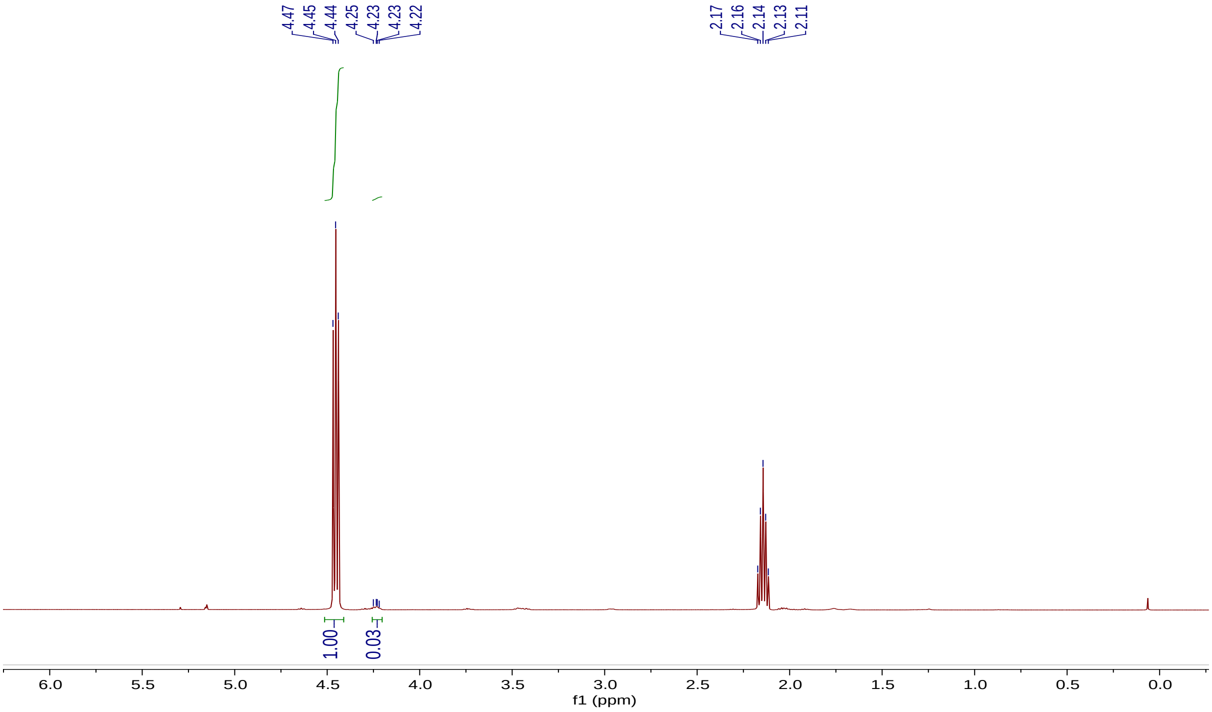

Table 3, entry 2

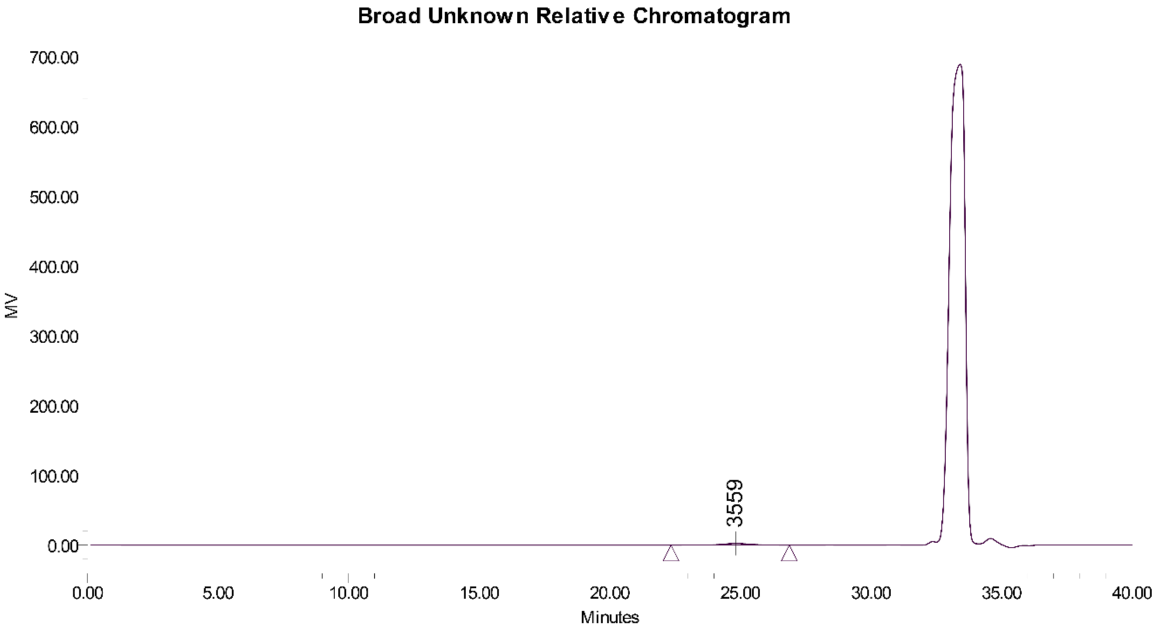

Broad Unknown Relative Peak Table

	Distribution Name	Mn (Daltons)	Mw (Daltons)	MP (Daltons)	Mz (Daltons)	Mz+1 (Daltons)	Polydispersity	Mz/Mw	Mz+1/Mw
1		3495	3775	3559	4137	4612	1.080123	1.095893	1.221949

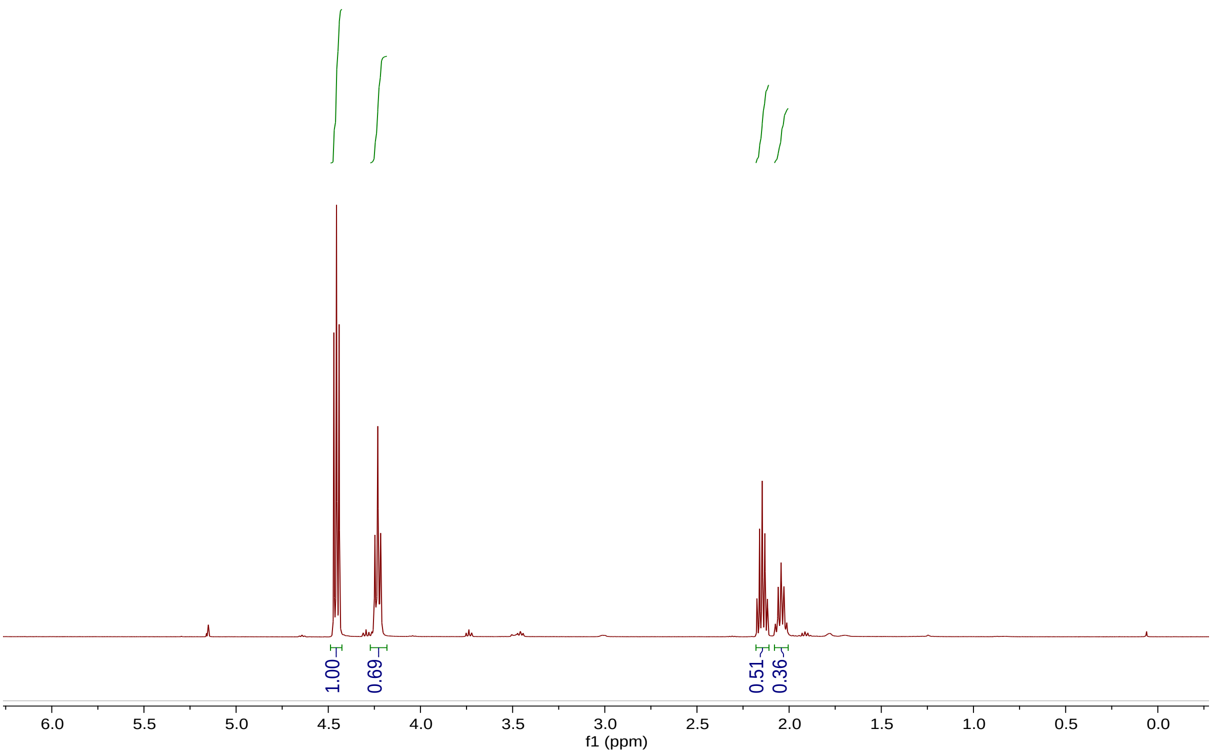

Table 3, entry 3

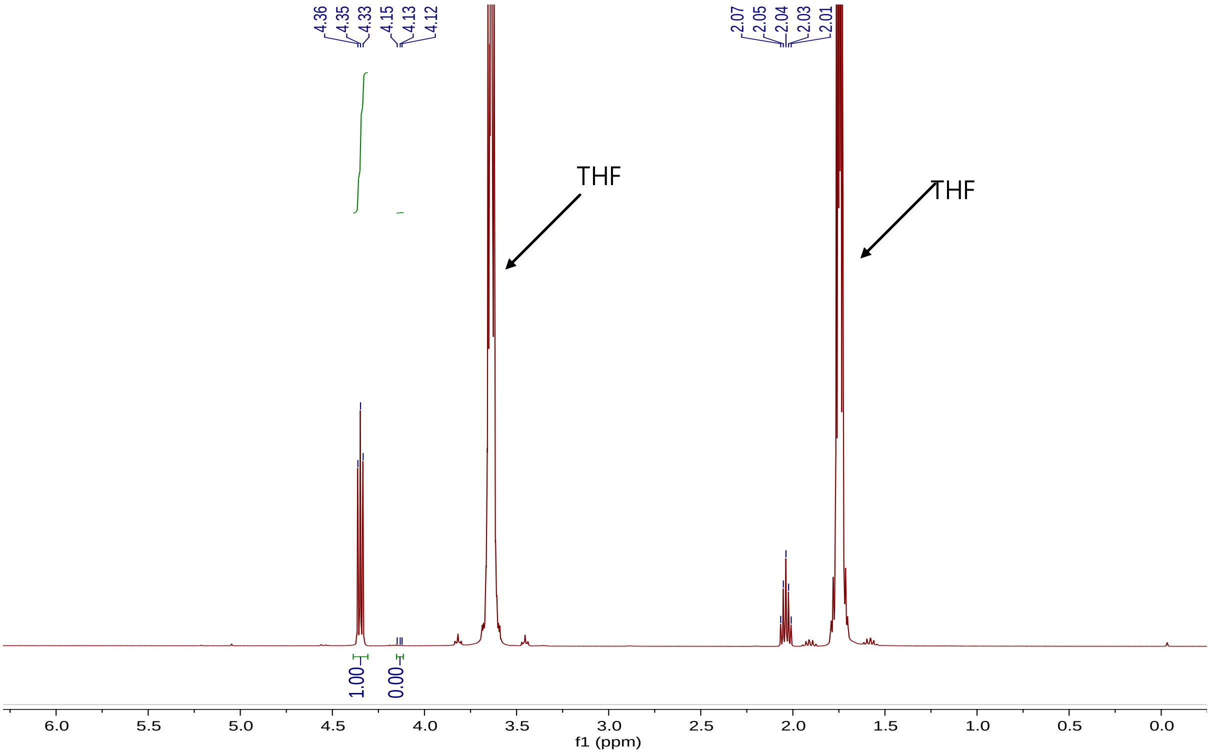

Table 3, entry 4

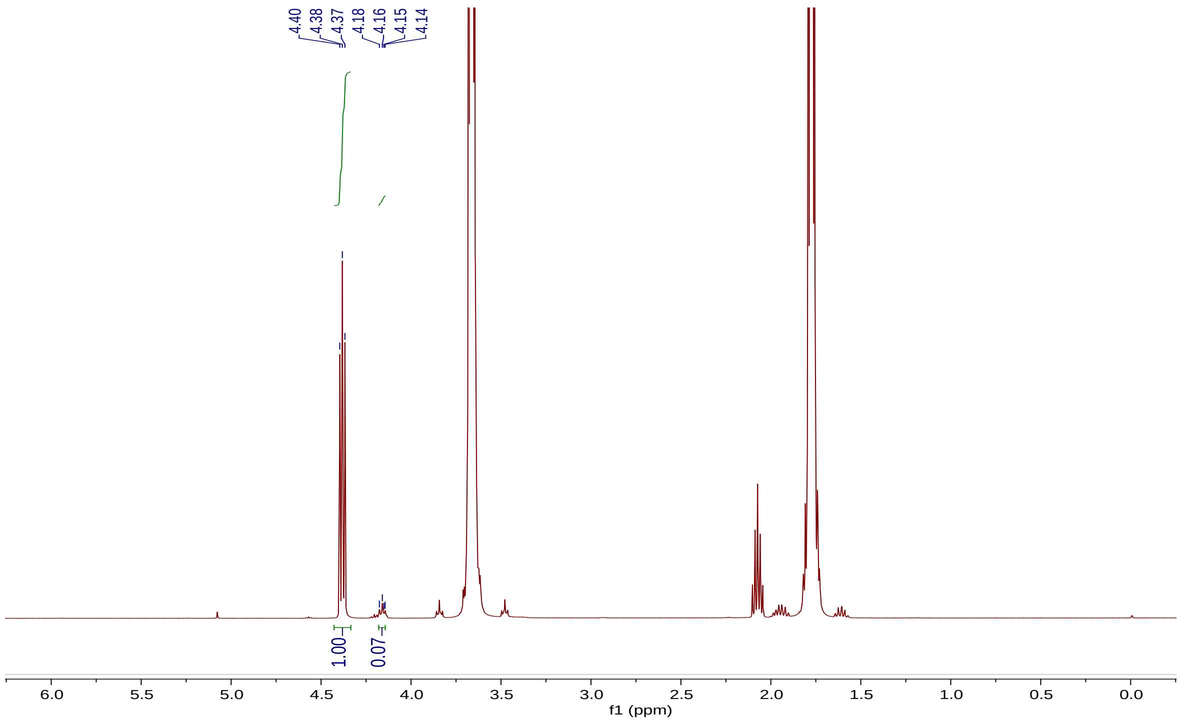

Table 3, entry 5

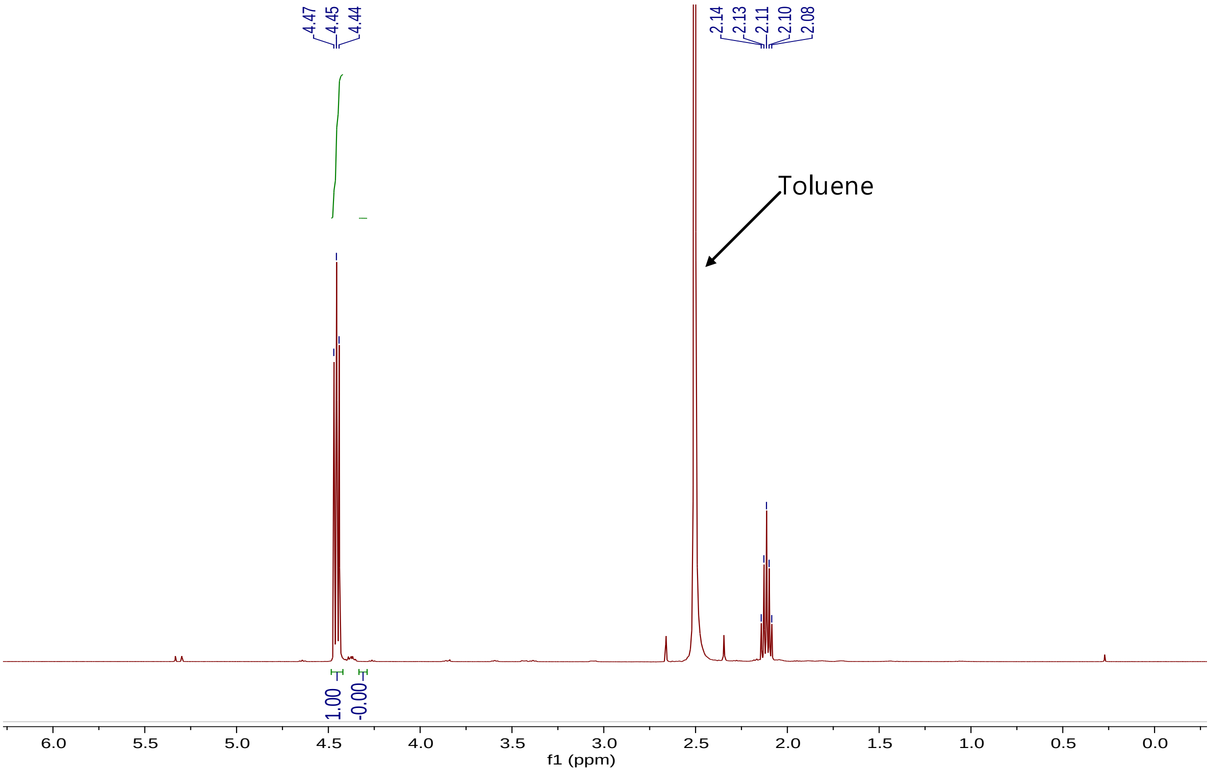

Table 3, entry 6

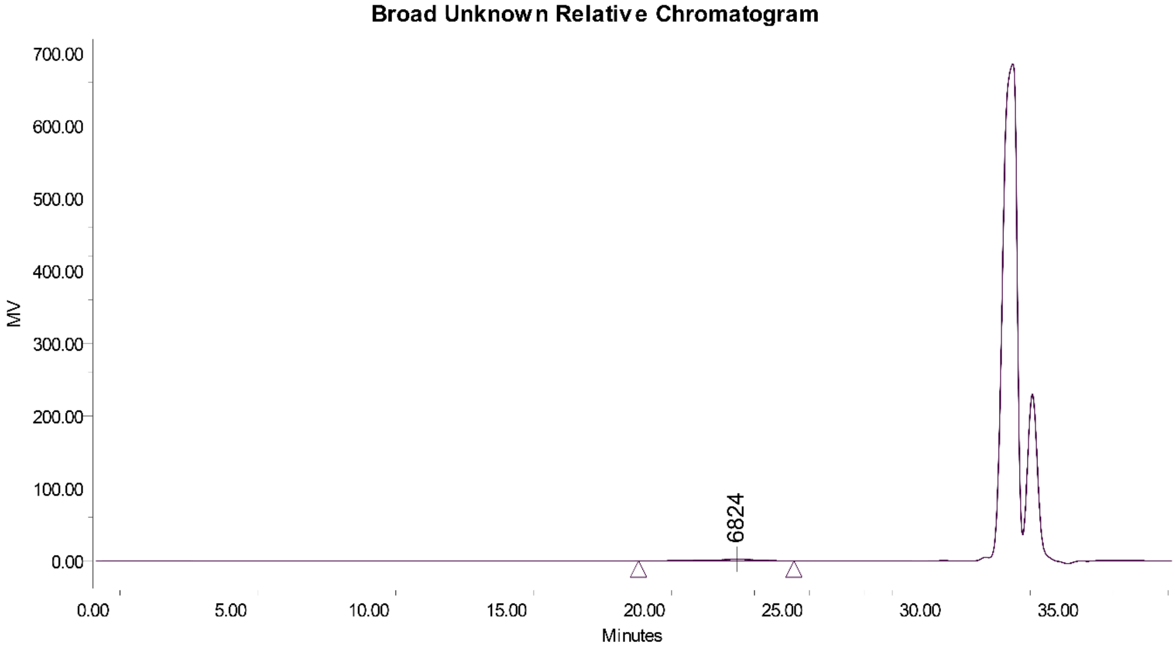

Broad Unknown Relative Peak Table

	Distribution Name	Mn (Daltons)	Mw (Daltons)	MP (Daltons)	Mz (Daltons)	Mz+1 (Daltons)	Polydispersity	Mz/Mw	Mz+1/Mw
1		7126	8376	6824	10337	13059	1.175459	1.234084	1.559024

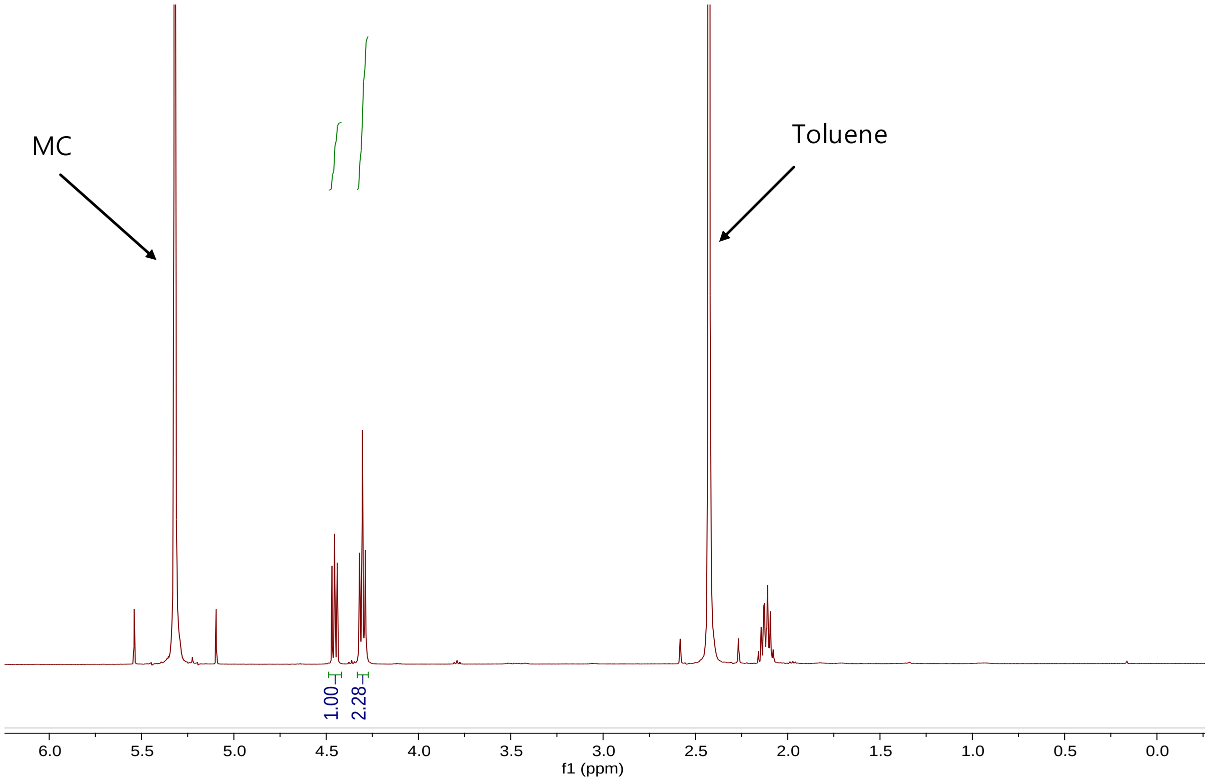

Table 3, entry 7

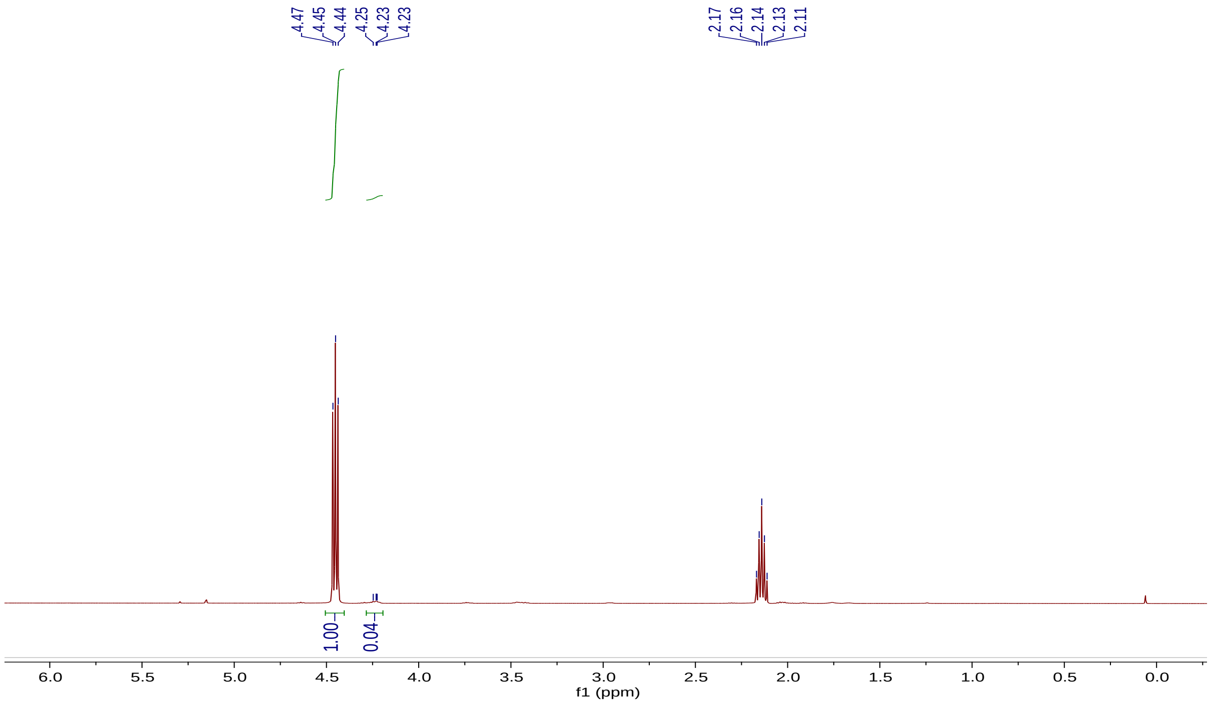

Table 3, entry 8

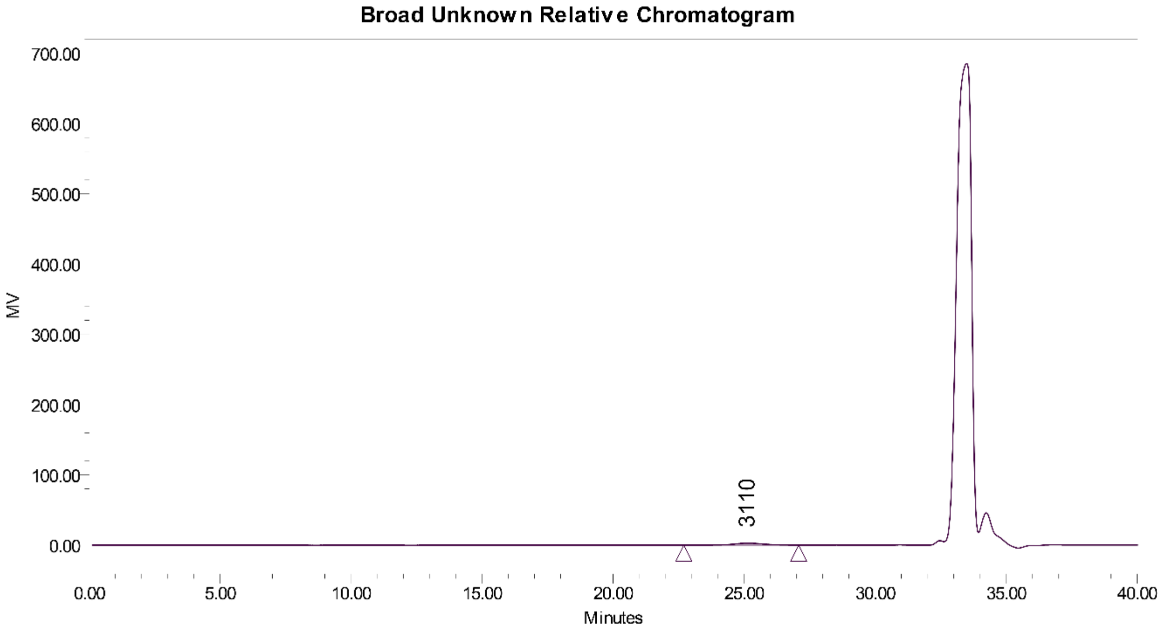

Broad Unknown Relative Peak Table

	Distribution Name	Mn (Daltons)	Mw (Daltons)	MP (Daltons)	Mz (Daltons)	Mz+1 (Daltons)	Polydispersity	Mz/Mw	Mz+1/Mw
1		3001	3209	3110	3468	3807	1.069408	1.080702	1.186363

4h

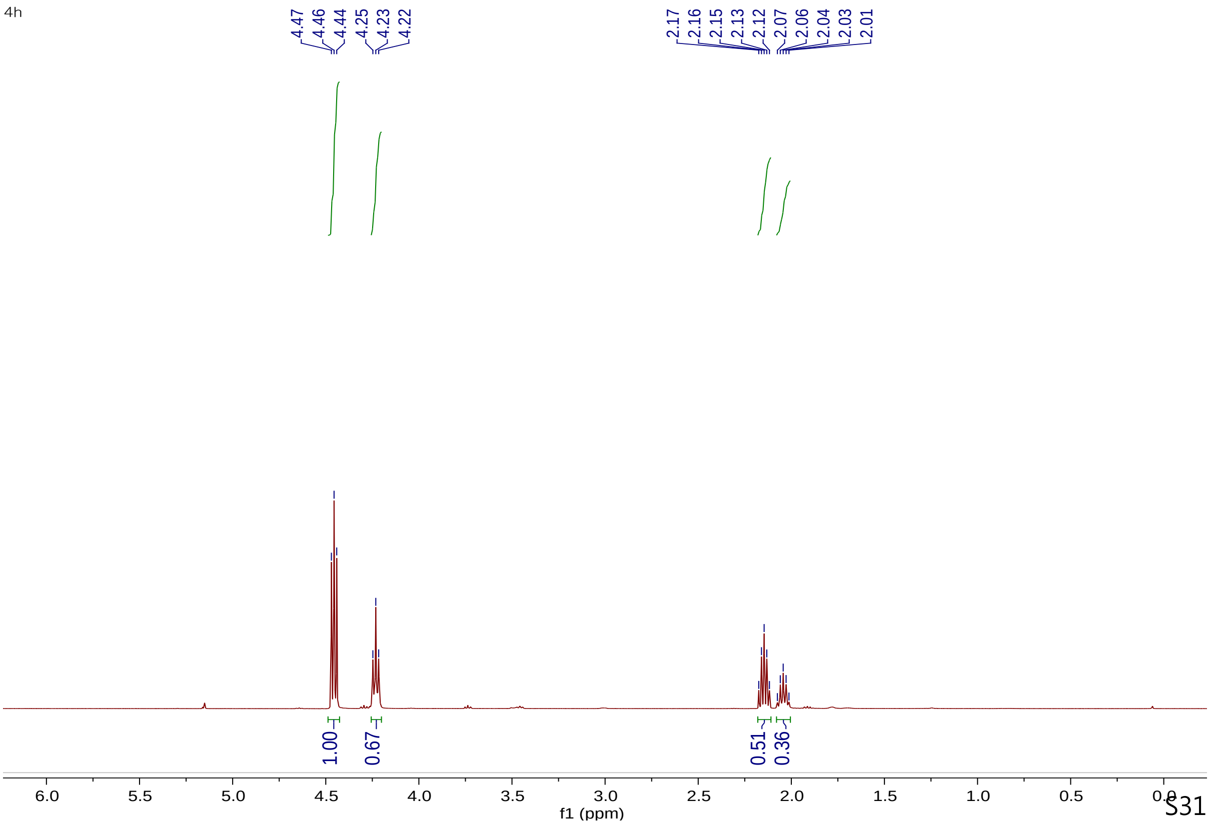

Table 4, entry 1

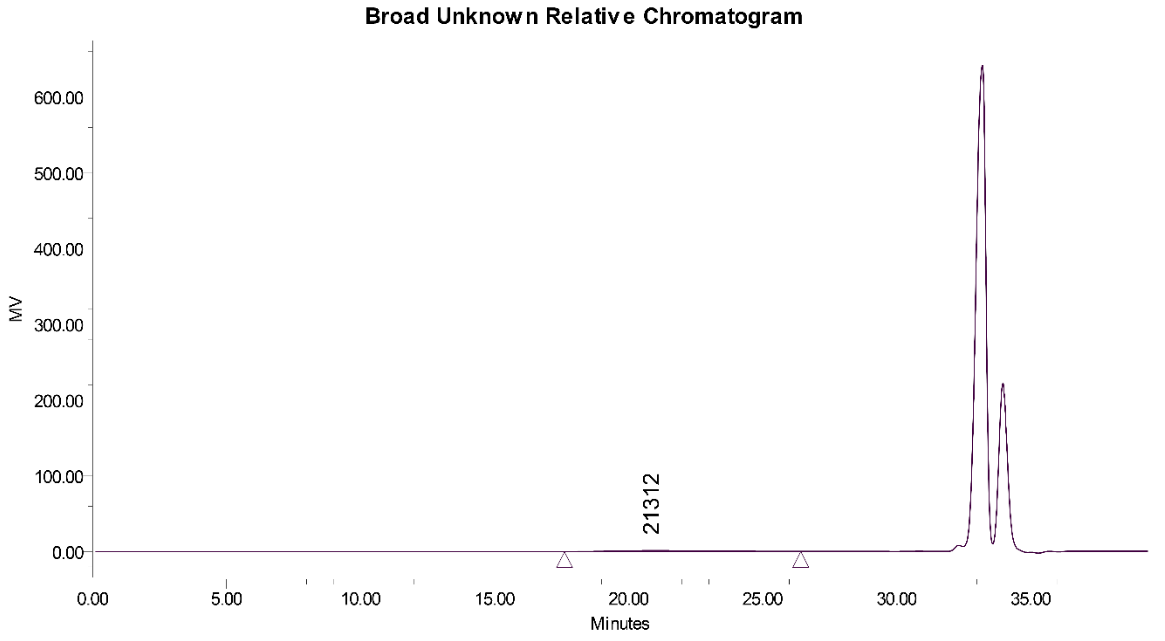

Broad Unknown Relative Peak Table

	Distribution Name	Mn (Daltons)	Mw (Daltons)	MP (Daltons)	Mz (Daltons)	Mz+1 (Daltons)	Polydispersity	Mz/Mw	Mz+1/Mw
1		12305	19975	21312	28465	36641	1.623306	1.425024	1.834316

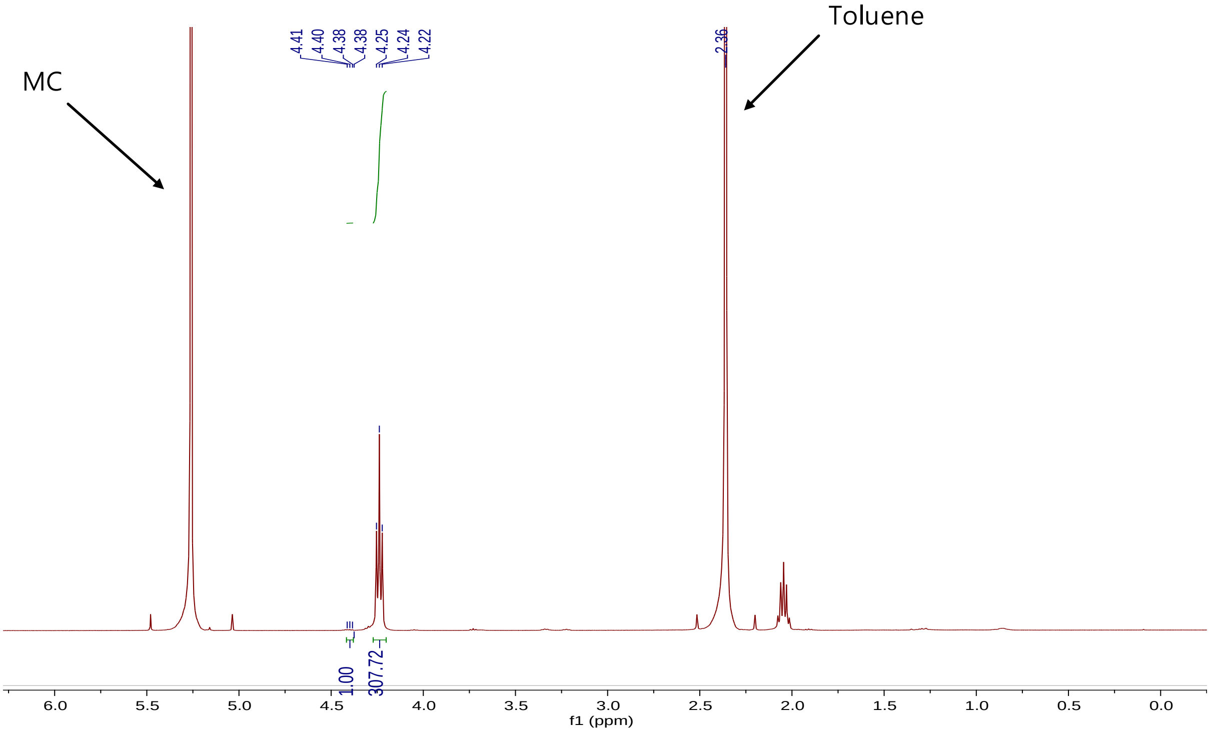

Table 4, entry 2

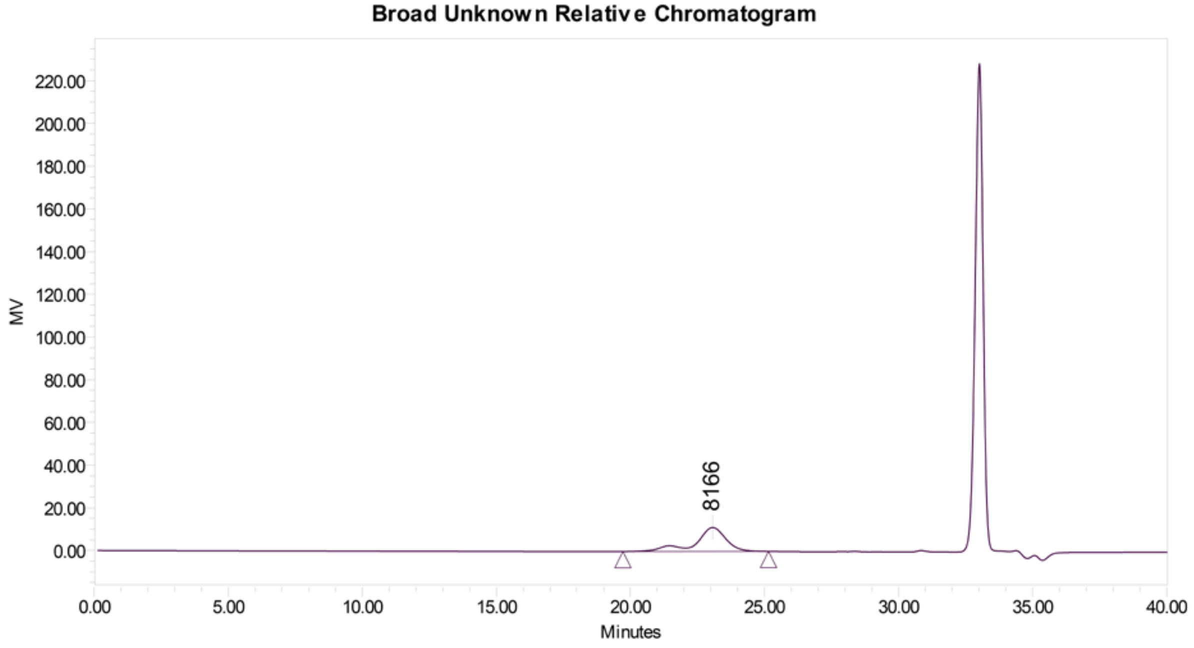

Broad Unknown Relative Peak Table

	Distribution Name	Mn (Daltons)	Mw (Daltons)	MP (Daltons)	Mz (Daltons)	Mz+1 (Daltons)	Polydispersity	Mz/Mw	Mz+1/Mw
1		8649	9808	8166	11507	13734	1.13411[illegible]	1.173128	1.400191

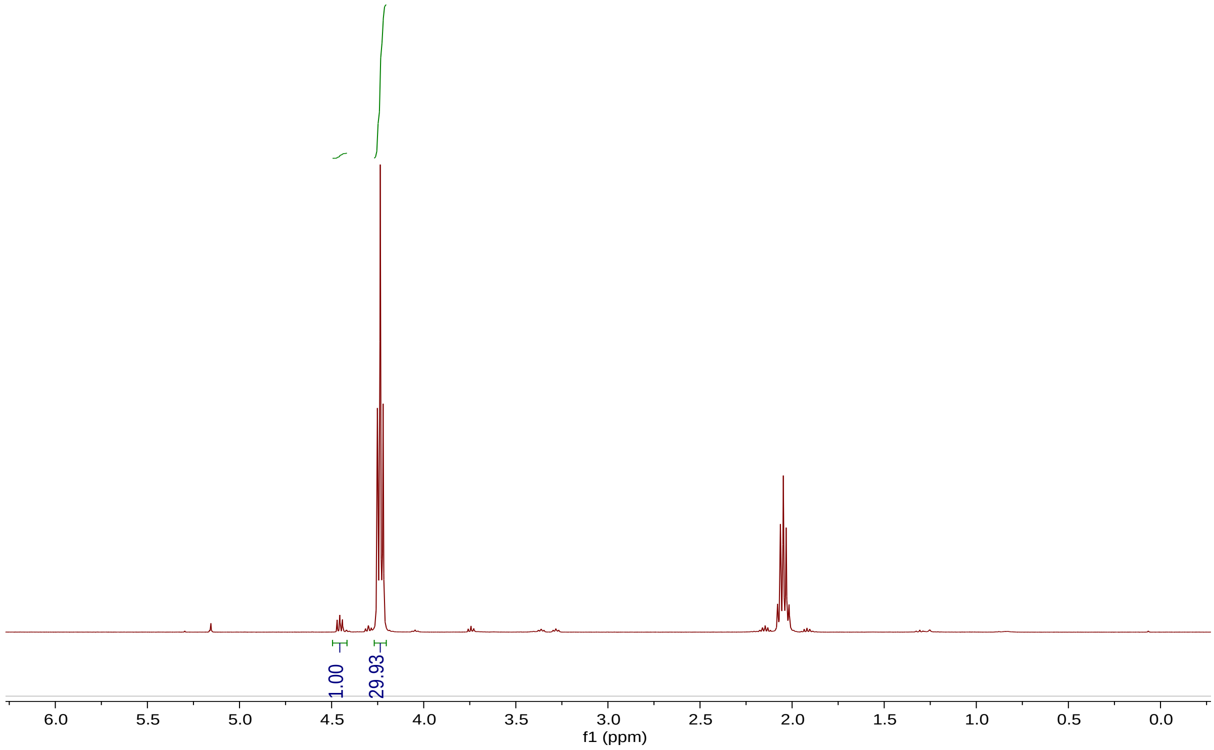

Table 4, entry 3

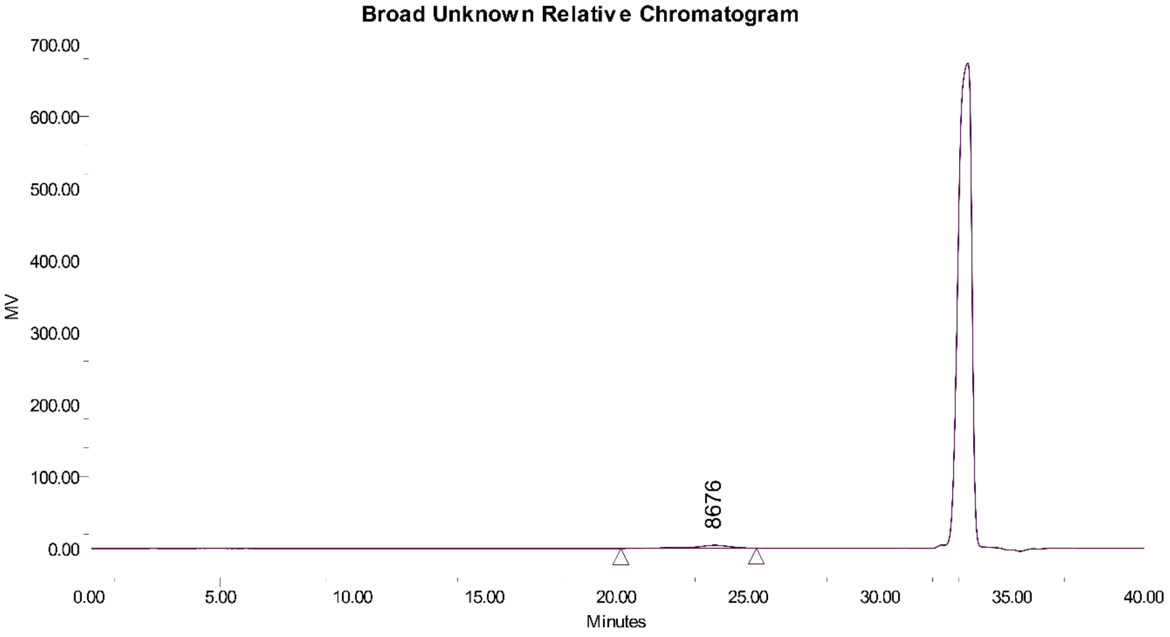

Broad Unknown Relative Peak Table

	Distribution Name	Mn (Daltons)	Mw (Daltons)	MP (Daltons)	Mz (Daltons)	Mz+1 (Daltons)	Polydispersity	Mz/Mw	Mz+1/Mw
1		9330	10527	8676	12204	14351	1.128225	1.159347	1.363303

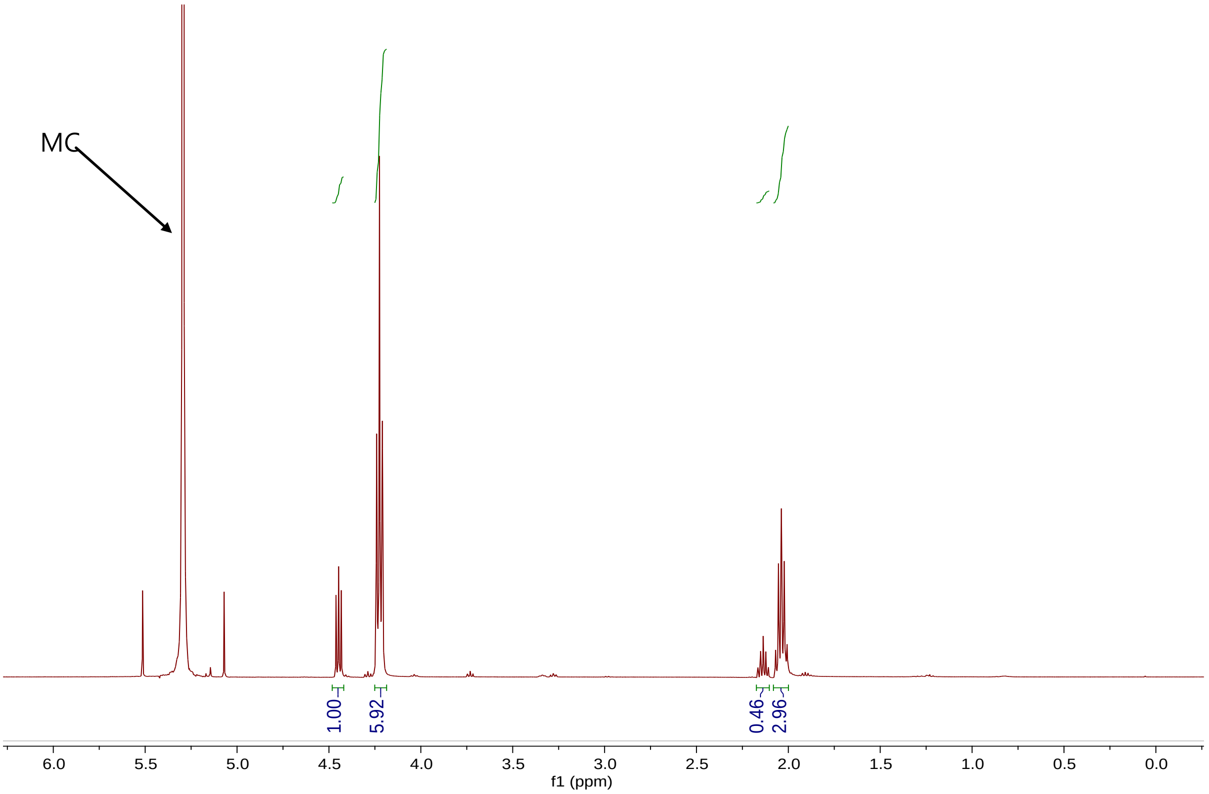

Table 4, entry 4

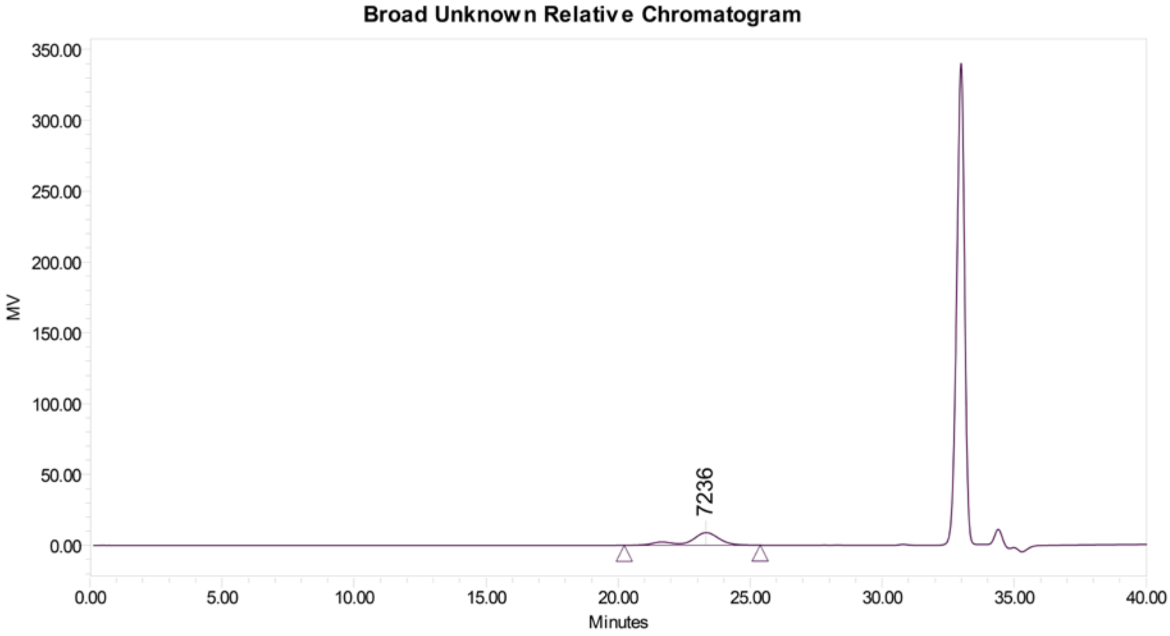

Broad Unknown Relative Peak Table

	Distribution Name	Mn (Daltons)	Mw (Daltons)	MP (Daltons)	Mz (Daltons)	Mz+1 (Daltons)	Polydispersity	Mz/Mw	Mz+1/Mw
1		7715	8750	7236	10246	12139	1.134142	1.171043	1.387370

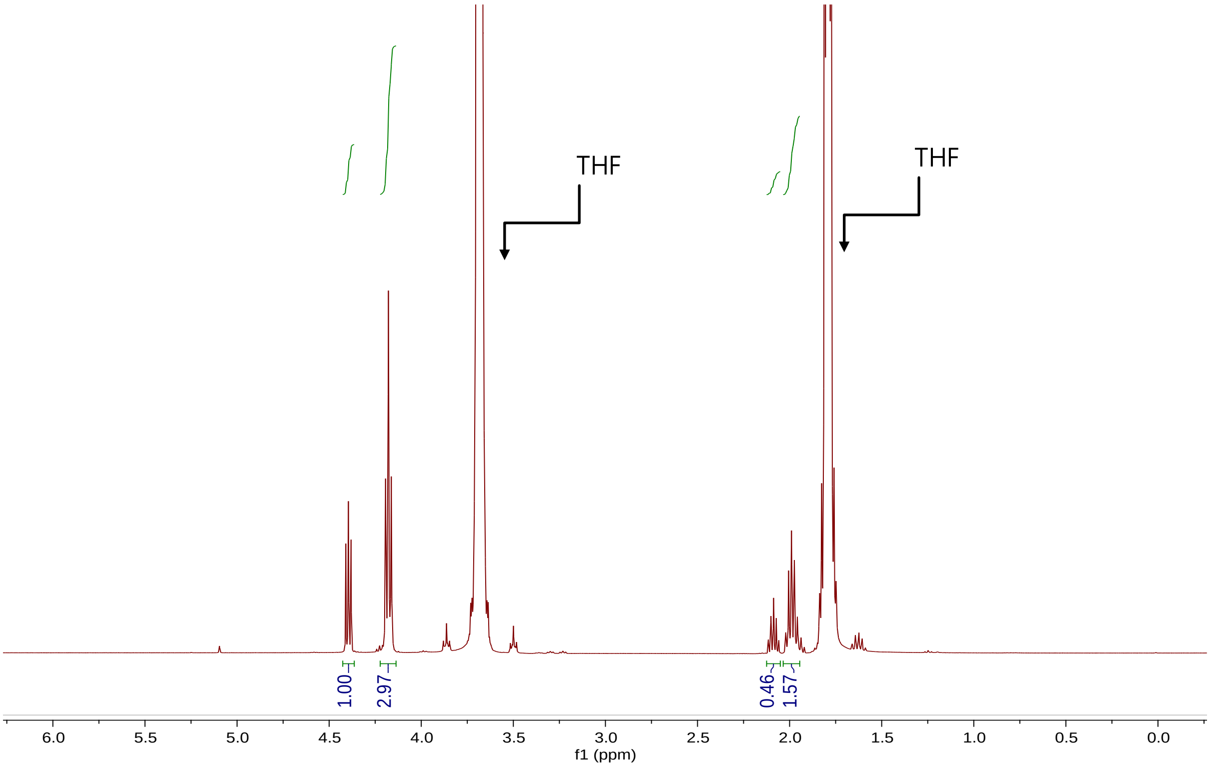

Table 4, entry 5 (Table S4, entry 5-1)

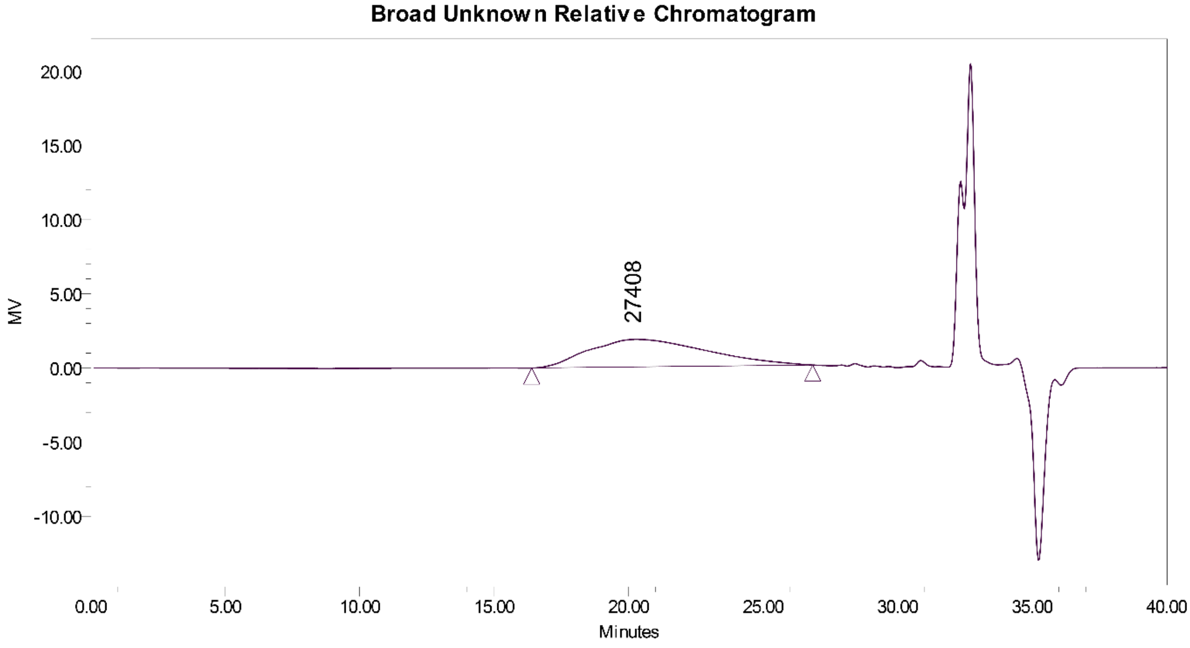

Broad Unknown Relative Peak Table

	Distribution Name	Mn (Daltons)	Mw (Daltons)	MP (Daltons)	Mz (Daltons)	Mz+1 (Daltons)	Polydispersity	Mz/Mw	Mz+1/Mw
1		13255	28811	27408	52460	80888	2.173500	1.820847	2.807549

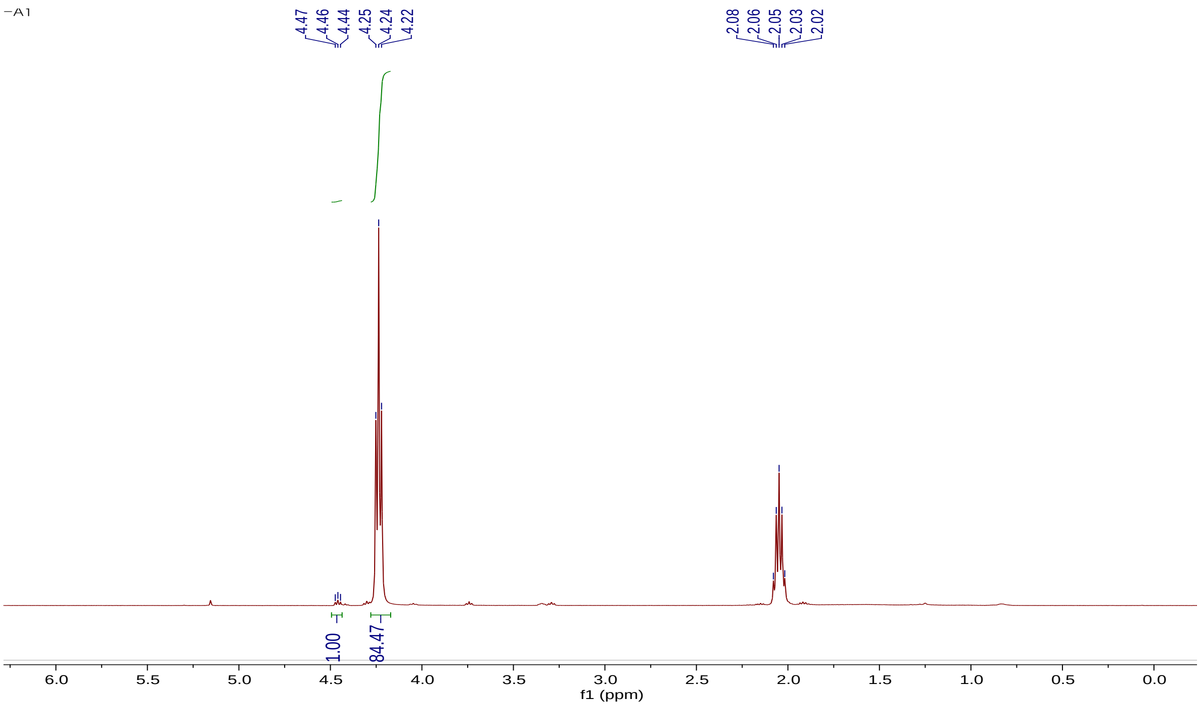

Table 5, entry 1 (Table S5, entry 1-1)

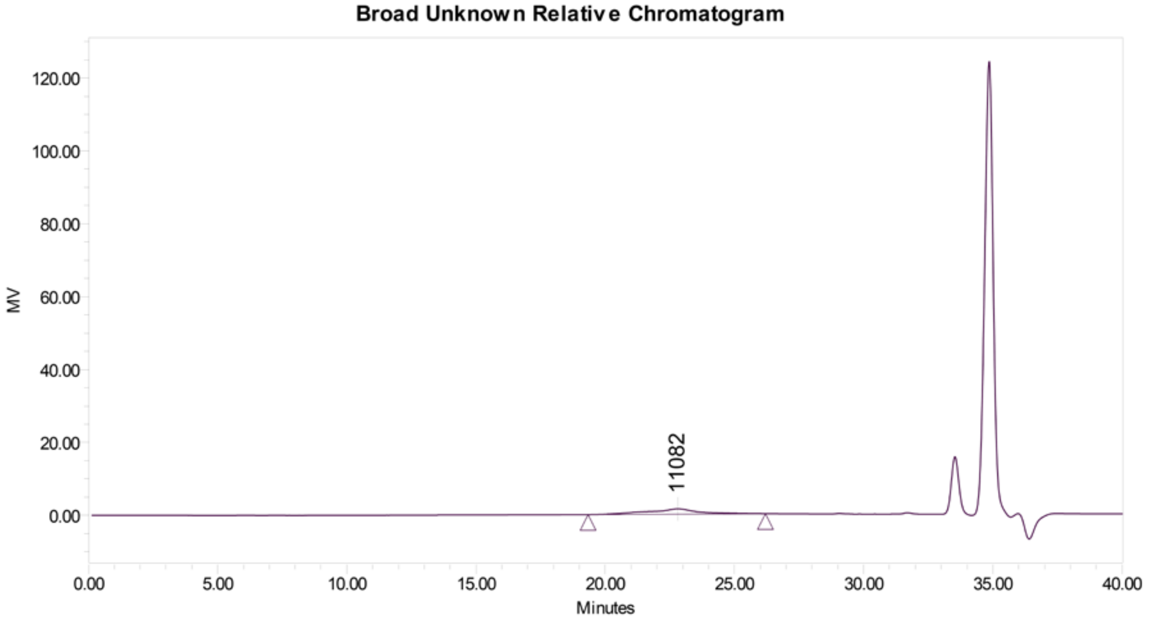

Broad Unknown Relative Peak Table

	Distribution Name	Mn (Daltons)	Mw (Daltons)	MP (Daltons)	Mz (Daltons)	Mz+1 (Daltons)	Polydispersity	Mz/Mw	Mz+1/Mw
1		10797	13691	11082	16951	20383	1.268072	1.238117	1.488795

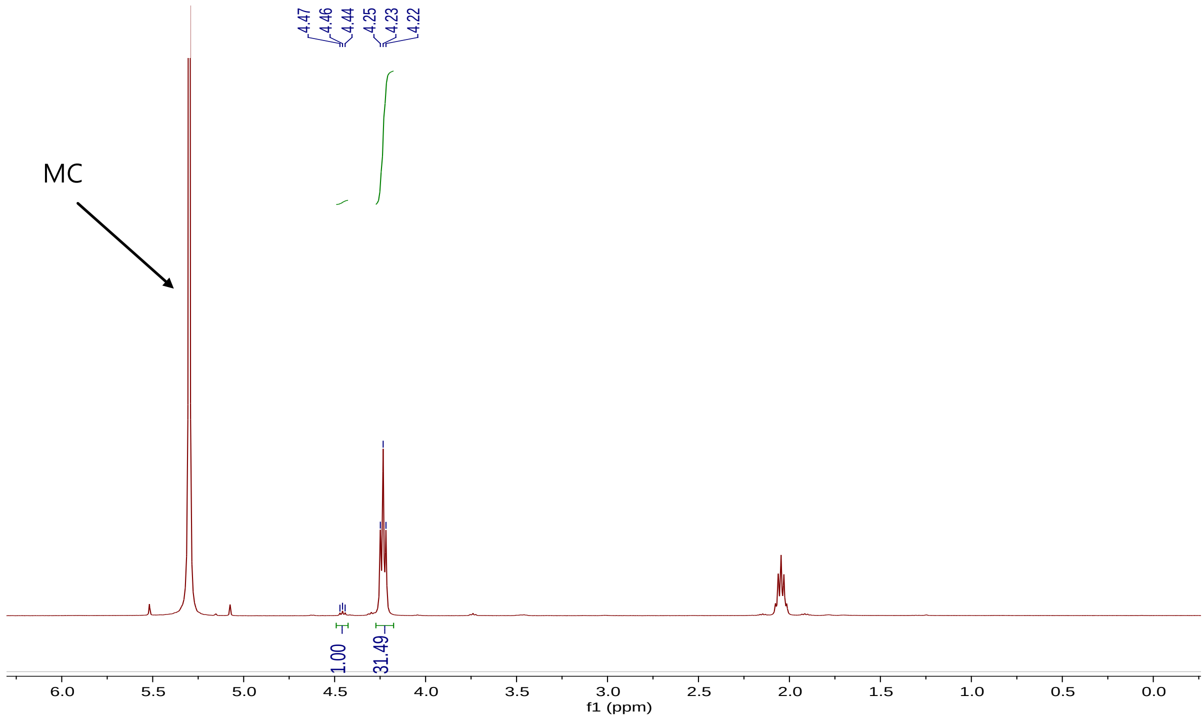

Table 5, entry 2

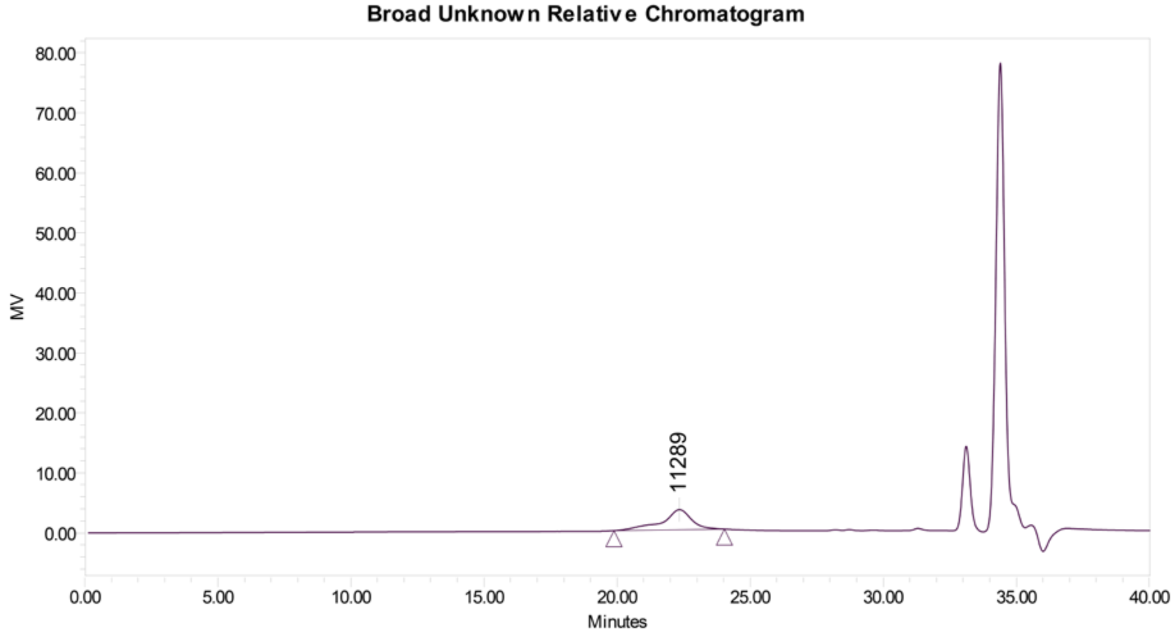

Broad Unknown Relative Peak Table

	Distribution Name	Mn (Daltons)	Mw (Daltons)	MP (Daltons)	Mz (Daltons)	Mz+1 (Daltons)	Polydispersity	Mz/Mw	Mz+1/Mw
1		11941	13094	1128[illegible]	14544	16273	1.096536	1.11074[illegible]	1.242804

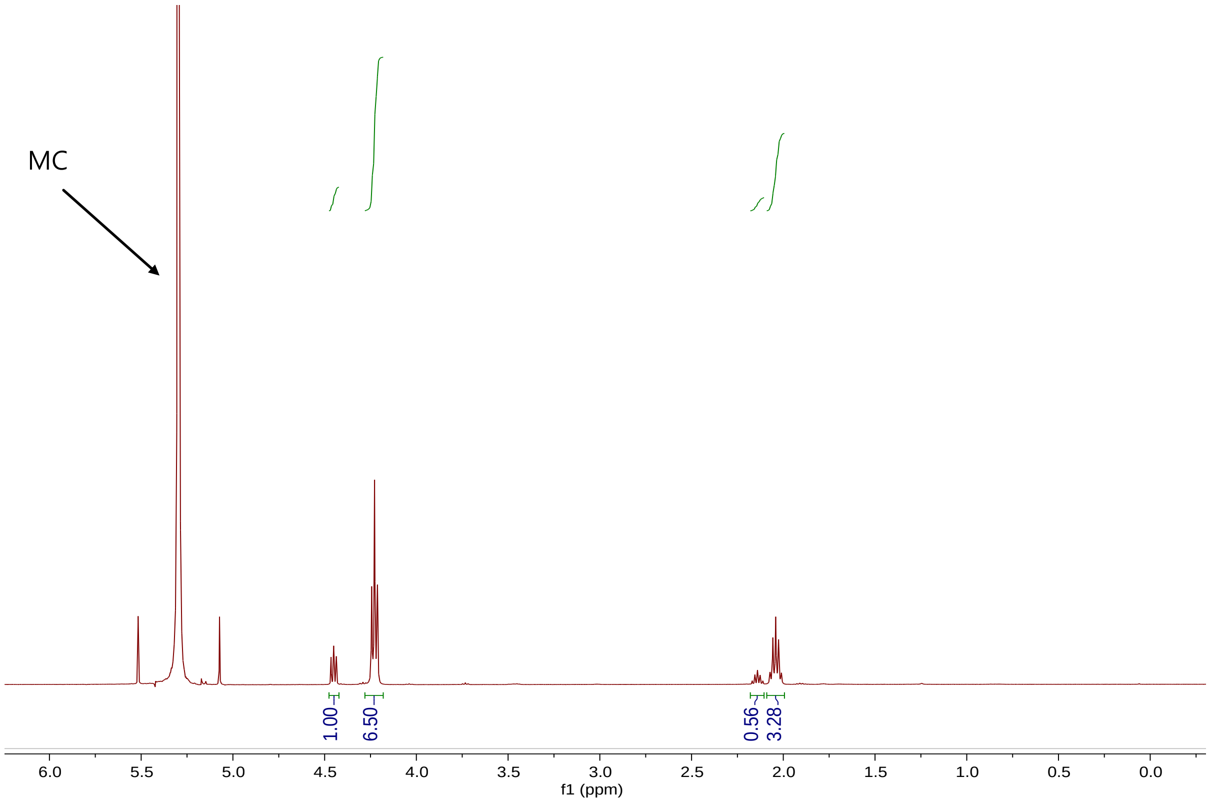

Table 5, entry 3

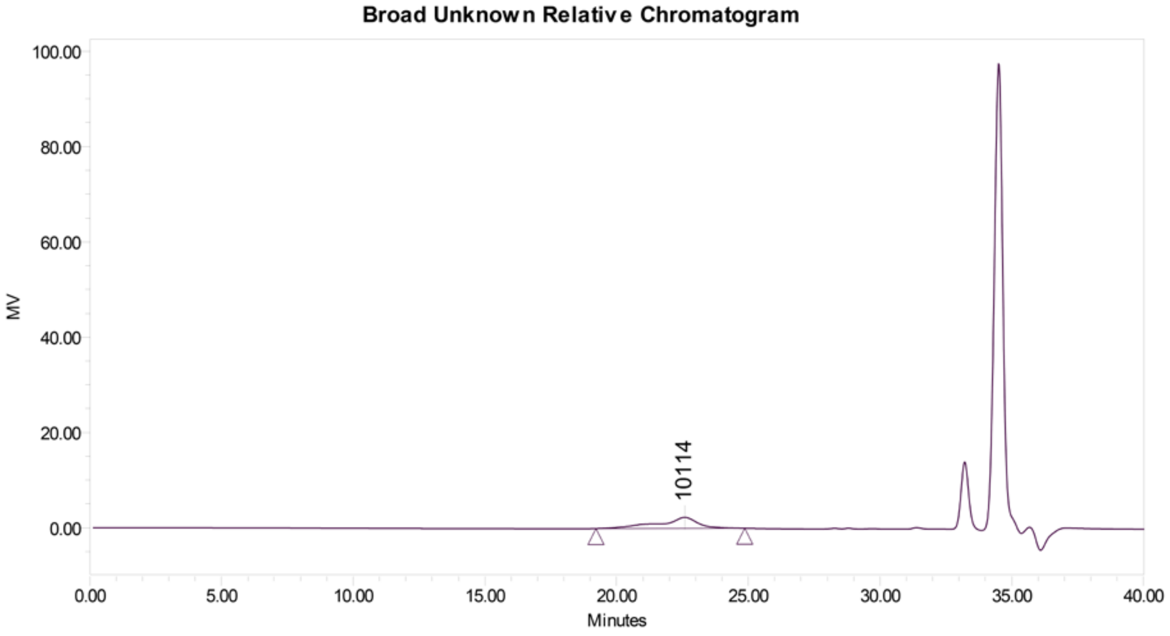

Broad Unknown Relative Peak Table

	Distribution Name	Mn (Daltons)	Mw (Daltons)	MP (Daltons)	Mz (Daltons)	Mz+1 (Daltons)	Polydispersity	Mz/Mw	Mz+1/Mw
1		11516	13689	10114	16527	19793	1.188658	1.207383	1.445949

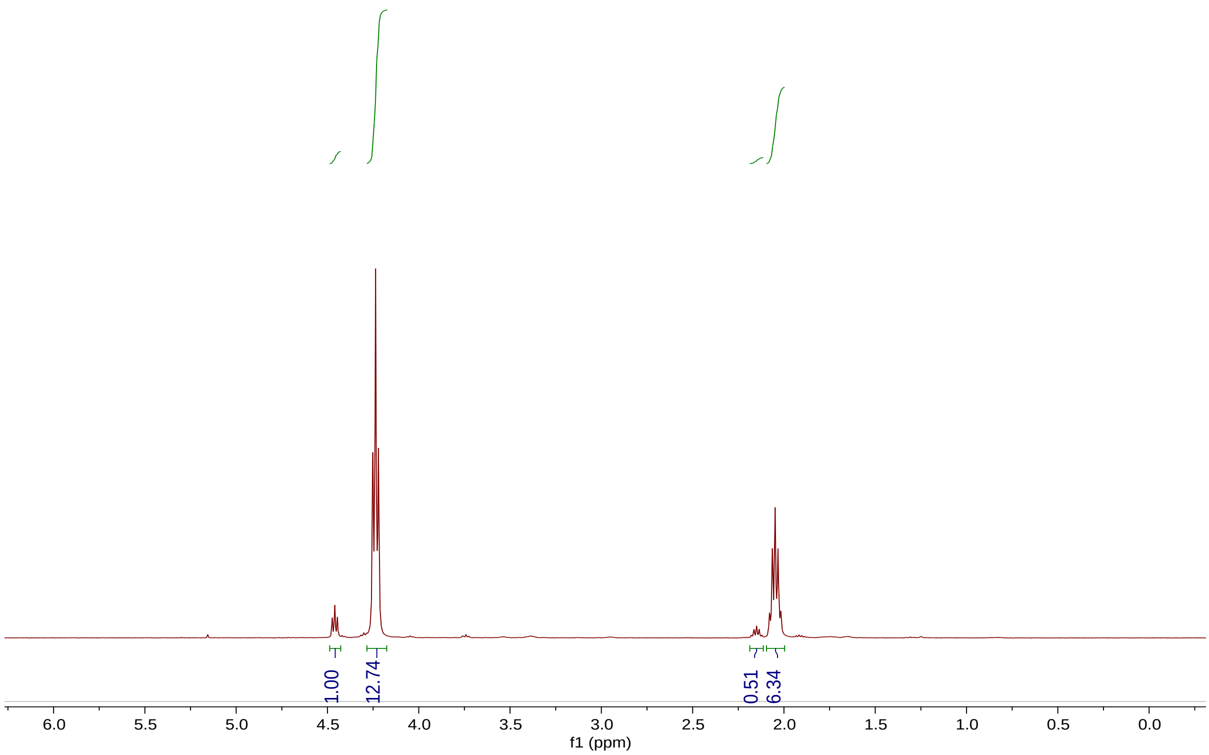

Table 5, entry 4

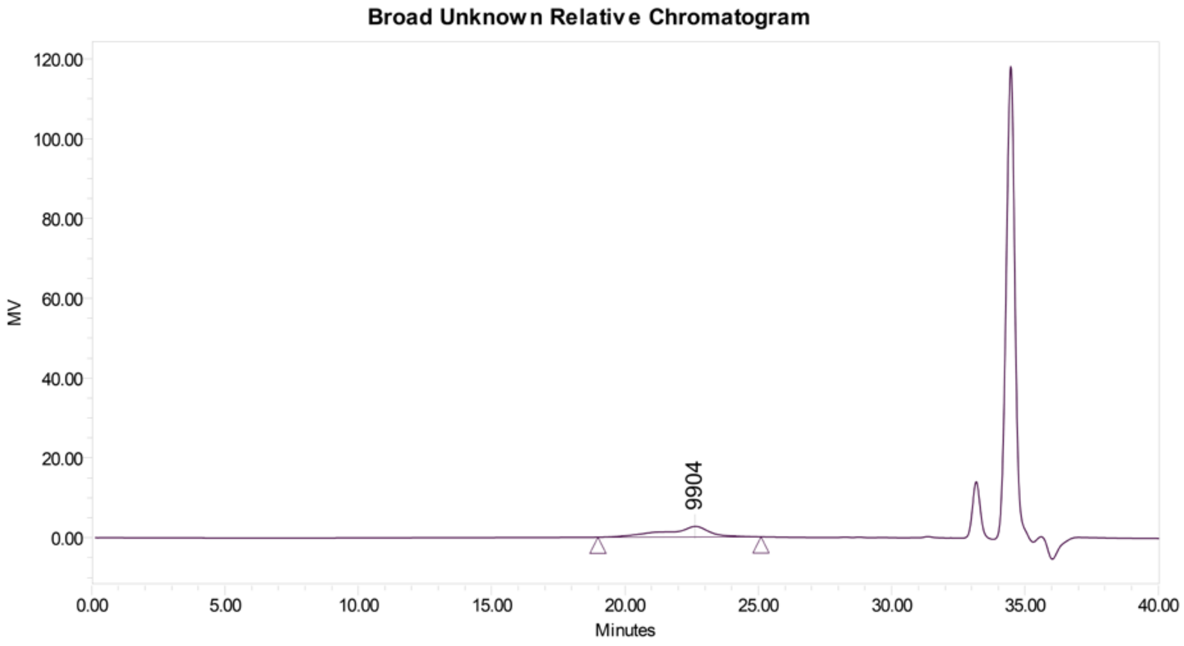

Broad Unknown Relative Peak Table

	Distribution Name	Mn (Daltons)	Mw (Daltons)	MP (Daltons)	Mz (Daltons)	Mz+1 (Daltons)	Polydispersity	Mz/Mw	Mz+1/Mw
1		1139[illegible]	13937	9904	17233	20976	1.222729	1.236503	1.505060

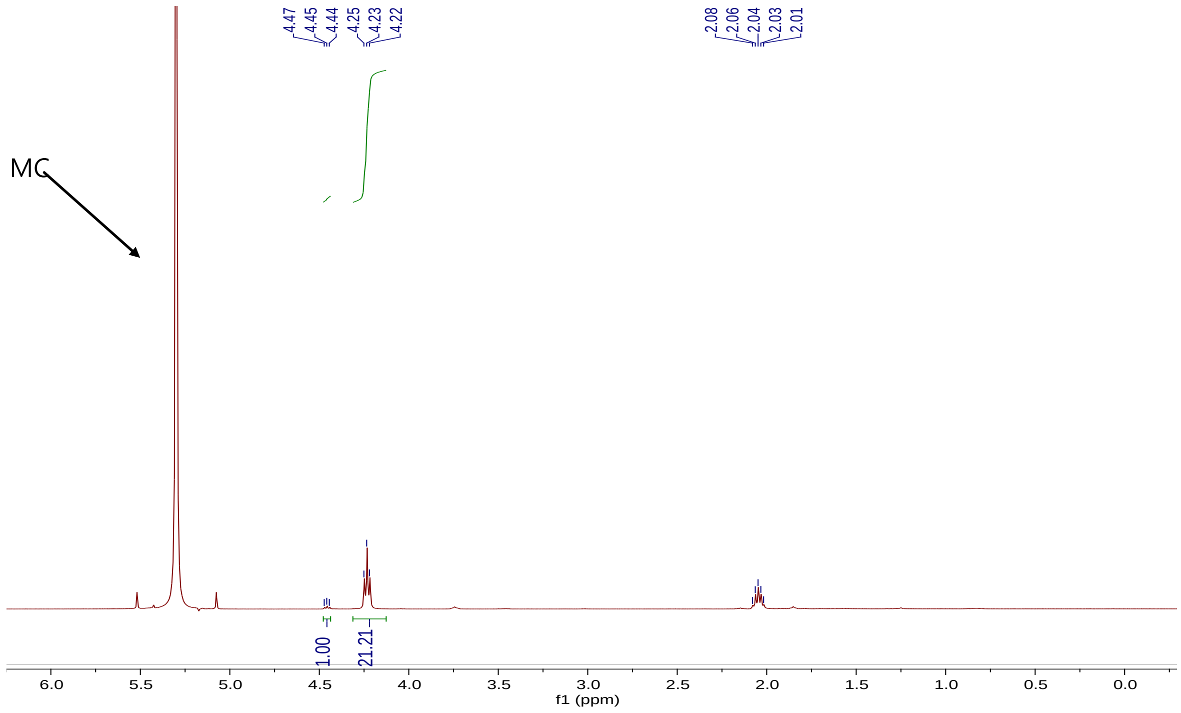

Supplement: File 1 — Raw data for tables, GPC and NMR spectra. [file Beilstein_J_Org_Chem-15-963-s001.pdf]
